# Leadership Development in Undergraduate Nursing Students: A Scoping Review

**DOI:** 10.3390/nursrep15050160

**Published:** 2025-05-02

**Authors:** Patrícia Costa, Joana Pereira Sousa, Tiago Nascimento, Paulo Cruchinho, Elisabete Nunes, Filomena Gaspar, Pedro Lucas

**Affiliations:** 1 Nursing Research Innovation and Development Centre of Lisbon (CIDNUR), Nursing School of Lisbon, 1600-190 Lisbon, Portugal; joana.sousa@ipleiria.pt (J.P.S.); tnascimento@esel.pt (T.N.); pjcruchinho@esel.pt (P.C.); enunes@esel.pt (E.N.); mfgaspar@esel.pt (F.G.); prlucas@esel.pt (P.L.); 2 Nursing Administration Department, Nursing School of Lisbon, 1600-190 Lisbon, Portugal; 3 School of Health Sciences, Polytechnic of Leiria, 2411-901 Leiria, Portugal; 4 Centre for Innovative Care and Health Technology–ciTechCare, 2414-016 Leiria, Portugal

**Keywords:** leadership, education, nursing, baccalaureate, nursing students, review

## Abstract

**Background:** Leadership is present at all levels of nursing and is essential to ensure the continuous improvement of nursing practice environments and the quality of the care provided to patients. This reality, coupled with the growing complexity of today’s health contexts, emphasises the importance of promoting the development of leadership skills in undergraduate nursing students, thus training nurses who are capable of acting as leaders and agents of change. To this end, a scoping review was carried out to map the available scientific evidence on the development of leadership in undergraduate nursing students. **Methods:** The scoping review was conducted according to two systematic review guidelines. The searches were conducted across a total of five databases for published studies and two databases for the unpublished/grey literature. The data extraction and analysis were performed by two reviewers, who independently screened and extracted data from the selected studies. **Results:** This review included 25 articles, and four thematic categories were identified—students’ perceptions of leadership; strategies to train leaders in nursing; the evaluation of leadership development; and conceptual models and curricula. The main conclusions highlight the need to reformulate existing curricula, the importance of integrating student-centred pedagogical approaches to promote leadership development, the impact that evaluating leadership development has on the whole process, contributing to the construction of an identity as a leader, and the need for it to be done in a structured and progressive way. **Conclusions:** The teaching of leadership should be promoted from the beginning of training, in a transversal, continuous, consistent, sustained, and articulated way, incorporating different disciplines, because only in this way will it be possible to train competent nurse leaders who are capable of acting in today’s complex and dynamic health contexts.

## 1. Introduction

Leadership has existed in human societies since ancient times [1], as evidenced by the numerous bibliographies on the subject. It is a complex and multidimensional concept that has been widely studied in various disciplines to better explain, understand, and define it [2,3].

In nursing, there has been a significant increase in research in this area in recent years, possibly due to the positive impact that leadership has had on the nursing practice environment (NPE) and the quality of care provided [4,5,6]. Several studies have shown that effective leadership styles are associated with higher levels of job satisfaction, productivity, staff retention, and an improved quality of care and satisfaction among the people being cared for, as well as a reduction in adverse effects, with lower rates of mortality and nosocomial infections [4,5,6,7,8,9]. For this reason, it is essential to identify which leadership skills are necessary for effective leadership and to understand how they can be developed [6]. Without adequate skills and knowledge, it is difficult for nursing leaders to maintain favourable practice environments [10,11], which in turn are associated with effective leadership styles, a higher quality and safety of care, and better outcomes for the people being cared for [12]. This proves that leadership influences the NPE and is strongly associated with a favourable NPE, constituting one of its dimensions [10,11].

In the last decade, several literature reviews have been carried out focusing on nursing leadership applied to nurse managers [13,14,15,16,17,18,19,20]. Regarding nurses, a few systematic reviews have been carried out which showed that leadership problems were related to the implementation of knowledge in practice, the quality of nursing care, and the availability of resources [21], and that the ability of nursing leaders to engage in change leadership in real-world contexts requires contextual evaluation [22]. Other systematic reviews have also found that nurses’ relational leadership styles are mediated by their ability to mobilise resources and achieve goals through access to information, support, and opportunities [23] and are also associated with higher levels of job satisfaction among staff [5]. Furthermore, another systematic review found that targeted educational interventions are an effective method for developing leadership skills in nurses [6].

A few scoping reviews have also been carried out, one of which explored the challenges and opportunities in developing the leadership of nursing and midwifery professionals [24], and another which showed that the competencies needed for clinical leadership are those produced by master’s-level training [25]. An integrative review revealed that nurses’ charismatic leadership is an important competence, especially in times of crisis and change [26].

Although leadership is a widely discussed concept, there is no universally accepted definition, which reinforces its complexity and great diversity [1,2,3,27]. For this reason, within the scope of this scoping review, we have chosen to define leadership in nursing as a social and relational process that takes place through positive influences and committed decision-making, which are reflected in actions and attitudes that benefit the people being cared for and the nursing and healthcare environments [1].

The diversity of the nursing profession is reflected in the different roles that nurses assume as an integral part of their professional practice, many of which occur simultaneously [2]. Leadership is present in all of them, from the care provided to the person being cared for to top-level decisions and health policies [6,28]. In this sense, it is crucial to promote the development of leadership skills in nursing, which should be worked on from undergraduate training [29,30,31,32,33,34].

Nurses, due to their training, competence, and comprehensive and insightful understanding of the people they care for, are particularly well placed to contribute to and lead changes in healthcare systems [2] and are increasingly expected to actively participate in all these processes [33]. This is foreseen in the *State of the World’s Nursing 2020* [33], which defined, as a strategic objective for 2030, the increase in leadership training to create more competent clinical nurse leaders [33,34] capable of directing transition processes, occupying strategic positions in teams (inside and outside them), and recognising the clinical results and associated costs, thus promoting improvements in the safety, effectiveness, efficiency, and quality of the care provided [35]. In addition, leadership has also been identified by the Canadian Association of Schools of Nursing [36] as one of the fundamental elements of undergraduate nursing training.

Leadership is seen as a competence that can be developed and improved, which is associated with behaviours and practices, not just personality traits [37], which highlights the importance and need to include it as a fundamental element from undergraduate nursing training [1,7,29,30,34,38,39,40,41]. Leadership development should be encouraged at every available opportunity, both inside and outside the classroom, with the aim of preparing nursing students for the future and fostering their career success [41]. This will promote the emergence of leadership opportunities in the settings where students later find themselves practicing their profession and prepare them to act effectively in today’s highly complex and challenging healthcare environment [2,41,42].

In recent years, different authors have focused their efforts on studying the development of leadership, with the aim of promoting it in the training of undergraduate nursing students. The results of these studies demonstrate the unequivocal importance of ensuring that this competence is properly developed throughout undergraduate training, which should be accompanied by an in-depth knowledge of the provision of care, the ability to work as part of a team, the ability to respond to complex needs with a view to improving quality, and a knowledge of economics, in order to become effective leaders in nursing [43].

In this sense, given the critical importance that leadership in nursing has for the NPE and for the quality and safety of the care provided, it is essential to recognise, identify, and explore how leadership can be promoted and developed in undergraduate nursing students to adequately prepare them for future challenges. However, although there is a consensus in the literature on the need to integrate leadership development into curricula, we believe that there are still gaps in the understanding of the processes involved and the most effective strategies for promoting it. For this reason, we chose to carry out a scoping review to map the available scientific evidence, identify gaps, and provide a solid basis for future educational interventions [44]. The choice to carry out a scoping review rather than a qualitative or mixed methods systematic review is based on the methodological guidelines established and recommended by the Joanna Briggs Institute (JBI) [45], which states that scoping reviews are particularly suitable for mapping key concepts within a topic, identifying gaps, and synthesising the available evidence, without restricting the analysis to studies with a specific design or strict quality criteria, as is the case with traditional systematic reviews [44].

In the context of developing leadership in undergraduate nursing students, the existing literature emphasises the importance of including this competence in curricula and encouraging every opportunity to consolidate it throughout training. However, there are still uncertainties about the most effective approaches to promoting its development, as well as which contexts are most favourable for its implementation and what results are expected, which underpins and justifies the need to carry out a comprehensive analysis of the subject.

In addition, qualitative or mixed-method systematic reviews are more suitable when there is a focused and well-defined research question, usually related to the effectiveness of a specific intervention or the experience of participants in a given context [46,47]. In the present case, the intention is not to evaluate the effectiveness of an isolated intervention, but rather to map and synthesise a scattered body of research that addresses different strategies, concepts, and empirical evidence on leadership development in undergraduate nursing students. This approach is consistent with the purpose of a scoping review, which makes it possible to identify research gaps, clarify concepts, and inform future systematic reviews on the subject [44].

To reinforce the need for this study, a search was carried out in the main review registry databases (PROSPERO, OSF Registries, INPLASY, and Cochrane Database of Systematic Reviews) and, to date, no completed or ongoing review was found with the aim of mapping scientific evidence on the development of leadership in undergraduate nursing students. This gap justifies the relevance and innovation of our study, as it proves that this topic has not yet been comprehensively reviewed.

Thus, this scoping review aims to map the available scientific evidence on leadership development in undergraduate nursing students, providing a more comprehensive view of the practices, approaches, and outcomes reported in the literature. With this, it is hoped that this review will contribute to a solid and practical knowledge base that supports the training of competent nurses capable of facing the complex and dynamic challenges that characterise today’s healthcare contexts.

## 2. Materials and Methods

This scoping review was conducted in accordance with the guidelines established by the JBI and reported according to the recommendations of the Preferred Reporting Items for Systematic Reviews and Meta-Analyses Extension for Scoping Reviews (PRISMA-ScR) [48]. The title was previously registered with the Open Science Framework (OSF) (https://osf.io/jm3nd/, accessed on 26 September 2024). The frameworks we used in this review were those of Arksey and O’Malley [49] and the JBI [45], which recommend a descriptive and thematic analysis of the data to answer the research question. We used meta-aggregation according to Lockwood et al. [50].

The review question formulated for this study was “How is leadership development characterised in undergraduate nursing students in the scientific evidence?”, which was elaborated based on the following PCC strategy: population (P), undergraduate nursing students from different years of training; concept (C), leadership development; context (C), nursing education. The aim of this review was to map the scientific evidence on leadership development in undergraduate nursing students.

### 2.1. Eligibility Criteria

The eligibility criteria were defined based on the PCC (participants, concept, context) mnemonic, according to the methodology proposed by the Joanna Briggs Institute (JBI) [48]:

- Participants: All articles focusing specifically on undergraduate nursing students from different years of training were included. All articles relating to students from training areas other than nursing or focusing on postgraduate, master’s, or doctoral nursing students were excluded.
- Concept: Articles that addressed the development of leadership competences in nursing were considered. Articles that did not explicitly address leadership development were excluded.
- Context: Articles were included from studies carried out in the field of nursing education, covering training programmes (curricular and extracurricular) in higher education institutions, practical contexts integrated into undergraduate nursing training, or theoretical–conceptual contributions. Studies carried out outside this educational context or in practical contexts associated with the professional development of nurses were excluded.

### 2.2. Types of Sources

This scoping review included primary studies (quantitative, qualitative, and mixed methods) and secondary studies (literature reviews and conceptual analyses), text and opinion papers, master’s and doctoral theses and dissertations, unpublished studies, books, and book chapters.

### 2.3. Search Strategy

To locate relevant studies, the research was structured in three sequential stages:

- First stage: An initial search was carried out in the CINAHL Ultimate (EBSCO) and MEDLINE Ultimate (EBSCO) databases to identify synonyms and variations of the search terms used to index articles, titles, abstracts, and keywords. The terms initially used were “nursing students”, “undergraduate students”, “leadership”, “leadership development”, and “nurse education”. Based on the terms identified, a complete search strategy was developed for each database (Supplementary File S1);
- Second stage: The previously defined search strategy was applied to the CINAHL Ultimate (EBSCO) and MEDLINE Ultimate (EBSCO) databases, focusing on titles. Subsequently, the search was expanded to the Scopus, ScienceDirect, and Web of Science Core Collection databases, using the same keywords and search terms, adjusted for each database, also at the title level. Additionally, the grey literature was searched in the LILACS and RCAAP databases, using the same search strategy and keywords;
- Third stage: The bibliographical references of the included articles were explored, and those that met the previously defined eligibility criteria for the review were added to the review. Articles recommended by experts were also included.

This research was carried out in October 2024 by two independent researchers and was restricted to full-text articles, which had abstracts and bibliographical references available, were open access, and were written in Portuguese, Spanish, and/or English—other languages were excluded due to the reviewers’ linguistic proficiency. The search also included the grey literature and unpublished studies.

As far as the time limit is concerned, articles published in the last 10 years (between 2014 and 2024) were considered, due to the updating of concepts and the greater development of the topic in this time frame.

### 2.4. Paper Selection

The search strategy led to a total of 677 records. Their references and citations were exported from the respective databases and imported into the Rayyan^®^ version 1.6.0. online platform to facilitate the management of the review. Using Rayyan^®^, duplicates were removed and the remaining records organised and screened according to the previously defined eligibility criteria. This process was carried out by two reviewers, and any differences were resolved by consensus, without the need to consult a third reviewer.

Thus, after eliminating duplicates, 566 records resulted. Of these, 42 were selected based on screening the titles and abstracts, followed by an analysis of the full text, which resulted in 14 reports being selected because they met the established eligibility criteria. The reference lists of these reports were scrutinised to identify potential additional articles of relevance and interest, based on the pertinence of their titles, resulting in the addition of seven records in accordance with the eligibility criteria. Four records were also included at the suggestion of experts.

In the end, 25 studies were included in this review.

**Figure 1:**
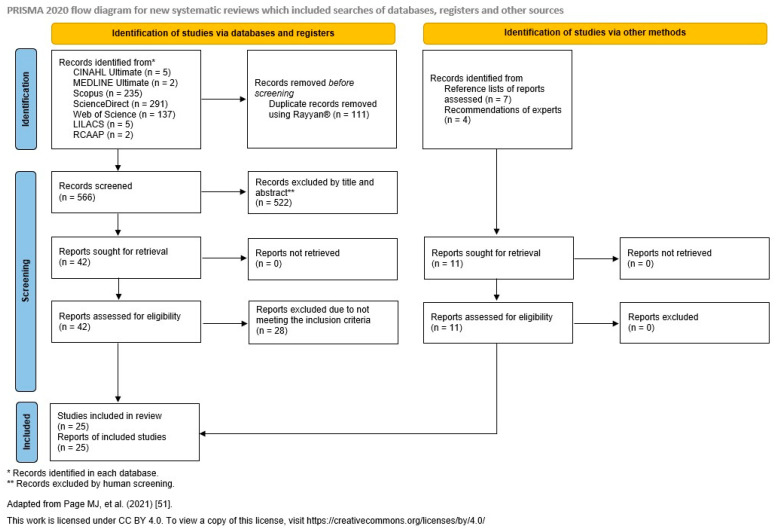
PRISMA 2020 flow diagram for new systematic reviews, which included searches of databases, registers, and other sources [51].

The process of the identification, screening, eligibility assessment, and inclusion of records is illustrated in the PRISMA 2020 flow diagram (Figure 1), according to Page et al. [51].

### 2.5. Data Extraction

The data were extracted by two independent reviewers using an extraction table specifically developed for the purpose, drawn up according to the review question and objective, as recommended by the JBI methodology [52]. In the end, the structure was organised as follows: author(s); year of publication; country; title; objectives; study design; study population/sample size/participants; context; relevant concept(s) of the review question/measurement instrument(s); and main results (Supplementary File S2). It was not necessary to contact the authors of the included articles to request additional or missing data.

## 3. Results

All the articles included in this scoping review were published between 2015 and 2024 and originated from different countries: the United States of America (16%; *n* = 4) [1,53,54,55]; Brazil (16%; *n* = 4) [38,56,57,58]; Spain (12%; *n* = 3) [27,29,59]; Australia (8%; *n* = 2) [60,61]; the United Kingdom (8%; *n* = 2) [62,63]; Turkey (8%; *n* = 2) [64,65]; Saudi Arabia (4%; *n* = 1) [66]; South Korea (4%; *n* = 1) [67]; Indonesia (4%; *n* = 1) [68]; Thailand (4%; *n* = 1) [69]; Taiwan (4%; *n* = 1) [70]; and Norway (4%; *n* = 1) [71]. There were also two articles resulting from international collaborations, namely between the United Kingdom and Israel (4%; *n* = 1) [72] and between Sweden and Jordan (4%; *n* = 1) [73].

The geographical analysis showed that most of the articles originated in countries outside the European continent (76%, *n* = 19) [1,38,53,54,55,56,57,58,60,61,64,65,66,67,68,69,70,72,73], specifically, there were eight articles from the American continent (32%) [1,38,53,54,55,56,57,58], seven from the Asian continent (28%) [64,65,66,67,68,69,70], six from the European continent (24%) [27,29,59,62,63,71], two from Oceania (8%) [60,61], and two from collaborations between countries located on two continents (Europe and Asia) (8%) [72,73].

Regarding scientific relevance, 22 of the articles were published in the last five years (88%) [1,27,29,38,53,54,55,56,57,58,59,62,63,64,65,66,67,68,69,70,71,72], and only three were published more than five years ago (12%) [60,61,73].

Most of the studies reported on qualitative research (36%; *n* = 9) [38,53,56,57,58,63,68,71,73], quantitative research (20%; *n* = 5) [27,64,67,69,72], or mixed-methodology research (12%; *n* = 3) [61,66,70]. In addition, three articles described intervention studies (12%) [54,55,59], two presented methodological studies (8%) [29,65], two reported studies of a theoretical–conceptual nature (8%) [1,60], and one documented a conceptual analysis (4%) [62].

Regarding the participants, the vast majority were undergraduate nursing students (*n* = 2054), representing 78.16% of the total. There were also 347 clinical nurses (13.20%), 168 nurse managers (6.39%), 57 academics (2.17%), and two newly graduated nurses who were preparing for the National Council Licensure Examination (NCLEX) for Registered Nurses (RN) (0.08%), who contributed and collaborated in the development of competences and leadership in undergraduate nursing students.

With regard to context, 19 articles were carried out in a university context (76%) [27,29,38,53,54,55,56,57,63,64,65,66,67,68,69,70,71,72,73], one in a hospital internship context (4%) [58], one in a health centre internship context (4%) [59], one corresponded to a national survey on curricular content in the field of leadership development (4%) [61], and three were carried out in a theoretical–conceptual context (12%) [1,60,62].

From reading and analysing the 25 articles included, four thematic categories emerged:

- Students’ perception of leadership;
- Strategies to train leaders in nursing;
- The evaluation of leadership development;
- Conceptual models and curricular programmes.

The identification of these categories was the result of a thematic analysis (Supplementary File S3), which was carried out in line with the aim of the review and according to a deductive approach (based on the contributions of the literature review previously carried out) and an inductive approach (based on the main patterns and themes identified when analysing the extracted data), as recommended by Peters et al. [48] and Pollock et al. [52]. Table 1 summarises the contribution of the 25 articles included to the four categories identified.

## 4. Discussion

The aim of this scoping review was to map scientific evidence on the development of leadership in undergraduate nursing students. To achieve this objective, 25 articles were included in the review, the analysis of which enabled four main thematic categories to be identified: (1) students’ perceptions of leadership; (2) strategies for developing leaders in nursing; (3) the evaluation of leadership development; and (4) conceptual models and curricula. We believe that these categories cover the fundamental dimensions of the leadership development process in undergraduate nursing students, whose understanding is indispensable and crucial for planning and implementing targeted, consistent, and effective educational interventions in this area.

### 4.1. Students’ Perceptions of Leadership: How Students Understand Leadership and Its Development

Leadership was widely recognised by the students as an essential skill for nursing practice [38,56,61,72,73], and the students also recognised that it directly influences the NPE and the quality and safety of the care provided, as well as the resolution of clinical complications, satisfaction, motivation, organisational commitment, teamwork, and the creation of role models for others [38,73]. In addition, they also identified that ineffective leadership leads to errors and adverse effects, which have a direct impact on the safety of the people being cared for and on health outcomes, and which, in turn, can be easily minimised through the exercise of effective leadership [38].

However, despite the consensus on its importance, most students reported not feeling prepared to take on this role [38,72]. This was clearly shown in Aydogdu’s study [38], where 70% of students admitted that they did not feel prepared to lead nursing teams in the future and recognised the urgent need to develop skills in this area. Similarly, Baron et al. [72] also highlighted this gap, pointing out that although in their study most of the students analysed reported being involved in leadership behaviours, they warned of the importance of ensuring a more consistent and structured application of these behaviours throughout their undergraduate training, to promote a solid and consistent development of these behaviours.

According to the study by James et al. [63], leadership development is closely related to the experiences and learning that students develop throughout their training, which directly influence, in a positive or negative way, their perceptions and attitudes towards the role of leader. Their results showed that students’ expectations and self-image were not always aligned with the image of effective leaders and that negative experiences generated concerns and feelings of not being prepared to lead [63]. These findings are worrying and reinforce the importance of promoting the proper development of a leader’s identity, since, as Miles and Scott [1] state, “possession of the requisite skills to lead is of no value if an individual does not perceive leadership as a critical dimension of nursing practice or if the individual does not label themselves a leader” (p. 8). In this context, the construction of a leadership identity is a crucial factor, since students need to recognise themselves as leaders so that they can exercise this role effectively [1,57,63,73]. According to these authors, this construction is underpinned by positive experiences, which promote self-confidence, and by the alignment between the process of developing competences and the perception of this competence as an integral and crucial part of professional practice [57,63,73].

Access to leadership opportunities and experiences was identified as a differentiating factor for its development, as described by Melo et al. [57] and Paim et al. [58]. In fact, in both studies, the students considered that the process of acquiring this skill depends on the opportunities to experience it in their practical contexts/internships, which, in turn, were identified as fundamental elements for the success of this whole process. However, in the study by Melo et al. [57], the very condition of being a student was pointed out as a significant limitation, being perceived as a barrier that hinders access to these same opportunities, especially when the practical contexts are highly demanding, as is the case with hospital emergencies. This jeopardises learning the role of a leader and, consequently, the leadership development process itself, intensifying feelings of fear and insecurity [57]. Allied to this, the scarcity of opportunities and inadequate conditions of some internship/clinical teaching contexts, namely contexts that are very focused on technical procedures/skills, have also been identified as barriers to the development of this type of skill, since, on the one hand, they further restrict access to leadership experiences, and on the other, they make it difficult to integrate a broader and more strategic view of nursing practice, which is essential for exercising effective leadership [57].

With regard to the competences needed to lead, Aydogdu’s study [38] showed that students recognised three main dimensions: (1) technical (procedural) competences; (2) human competences (which include effective communication, promoting environments that motivate the team, resolving conflicts, and defining common objectives); and (3) conceptual competences (for understanding and analysing situations that are considered complex). In the area of competences, Jack et al. [63] corroborate these statements, but go further, introducing the concept of clinical leadership and concluding that this is shaped and influenced by a combination of factors, namely interpersonal skills, up-to-date clinical knowledge, courage, confidence, the ability to adapt to change, the ability to work as part of a team, and the ability to become a role model for others.

The transition from theory to practice emerges from analysing the articles included as a critical aspect. In this sense, clinical leadership is perceived by students as an essential tool, playing a central role in integrating theoretical knowledge into practice [58,61,73]. In the study by Démeh and Rosengren [73], students identified clinical leadership as a source of security and a means of bridging gaps between academic training and clinical practice to promote safer and more effective care. They therefore recognised it as a fundamental need, with proven benefits that go beyond the professional, as they considered that it contributes to personal development, increasing the ability to organise, define objectives, and priorities. For this reason, it is seen as a necessity and not an option, and its systematic promotion throughout undergraduate training is advocated [73]. Clinical leadership thus stands out as a differentiating factor that has the potential to transform clinical contexts and strengthen a leader’s identity [73].

All the articles included and analysed in this scoping review unanimously argue that the teaching of leadership in nursing should be promoted and integrated into undergraduate curricula from the earliest years and developed in a transversal, continuous, consistent, sustained, and articulated way, incorporating different disciplines [1,27,29,38,53,54,55,56,57,58,59,60,61,62,63,64,65,66,67,68,69,70,71,72,73] in a holistic manner [56]. However, despite this consensus, studies have identified a few challenges and areas for improvement for the effective and structured implementation of this objective.

One of the aspects most recommended by the participants was the renewal and updating of current curricula [38], which are excessively focused on technical skills/procedures [56]. The current approach has been strongly criticised as it is considered to limit the development of a holistic and strategic view of nursing practice, which is recognised as essential for training future leaders [56]. The relevance of this curricular gap is reinforced by Aydogdu’s study [38], in which 40 per cent of the students mentioned that they had not attended specific classes on leadership, despite having already completed more than half of their course. This result reflects a structural insufficiency in the curricula, which can significantly hinder leadership development. In this sense, it has been suggested and recommended that programmes should include more opportunities to promote leadership development, with the aim of strengthening the self-esteem and performance effectiveness of students in terms of strategic thinking, emotional intelligence, impact and influence, and teamwork skills [72].

The different studies analysed also agreed on the continuous articulation and integration of theory and practice, since both are essential in the development of leadership [58,63,73]. This aspect was particularly emphasised in the study by Paim et al. [58], which showed that the development of leadership in the students in question took place in concrete practical situations, duly contextualised and theoretically grounded, which proved to be essential for consolidating competences and building the identity of leaders.

Strengthening educational practices was also mentioned as a suggestion to be applied throughout the training programme, with the aim of guaranteeing a continuous, consistent approach that promotes solid development [38,56]. Within this context, the role of teachers was highlighted, as they were recognised by the students as being responsible for preparing them for leadership [61]. Teachers have been given the duty to identify and prioritise all the opportunities that make it possible to exercise leadership at all levels, so that students can practice and rehearse it in a safe and controlled environment [57,62]. This idea was also corroborated in the study by Melo et al. [57], whose results emphasised the importance of qualified intervention by teachers, whose actions highlight them as points of reference and as facilitators of the learning process, and who do not confine students to a passive or merely expectant position [57,61]. In addition, sharing their personal experiences was also identified as a valuable tool, from which students felt they could learn about the difficulties and challenges inherent in leadership through concrete examples, as well as better understand the application of theoretical concepts in practice, which will influence and support their future decision-making [56].

This perspective is in line with the conclusions of Jack et al. [62], who argue that a teacher should adopt a welcoming and supportive attitude, as this is the only way to promote self-confidence and the capacity for autonomous learning in students [57,61,62,66]. In addition to the direct relationship with students, the importance of effective collaboration between teachers and members of the healthcare team and the healthcare institutions themselves has also been emphasised, and this collaboration has been identified as one of the contributors and determining factors in creating positive learning experiences [73].

Still, in the context of strengthening educational practices, there are also recommendations aimed at methodological approaches, pointing to the use of methodologies that are innovative and capable of making the teaching–learning process more dynamic and centred on leadership development. In this sense, the integration of diverse strategies is recommended, such as the use of educational videos [56]; practical workshops/theoretical-practical classes [56]; interactive forums [55]; themed events on leadership, such as congresses and conferences [38,56]; targeted theoretical approaches (with more specific classes) [38]; targeted courses [38]; simulated practices/realistic simulations [38,57]; role-playing [38]; and reflective practice [56].

In this context, it is imperative to explore and deepen effective pedagogical strategies to promote leadership development, and the following section will be dedicated to this purpose.

### 4.2. Strategies to Train Leaders in Nursing: Active Learning Methods and Educational Models

Many of the suggestions and recommendations presented in the previous section fit in with active learning methodologies. According to Calderon et al. [74], these methodologies involve the active participation of students, giving them greater responsibility for their own learning process. This has been shown to be associated with higher levels of motivation and satisfaction, resulting in an active construction of knowledge [74]. In these approaches, students develop competences through activities proposed and guided by teachers, which are carried out using collaborative and cooperative learning strategies between two or more students [74]. The teacher’s role is that of supervisor, responsible for proposing discussions, reflections, and challenges, acting as a facilitator of learning, rather than the traditional role of the transmitter of knowledge [27,54,57,64,66,74].

Various methodologies and pedagogical approaches can be used in the context of developing competences. However, within the scope of this scoping review, those that have been shown to have proven benefits in the development of leadership in undergraduate nursing students were identified:

- Tutoring/mentoring:
  In Bright’s study [53], tutoring/mentoring between students at different levels of education was associated with the development of leadership skills, with significant improvements in the areas of communication, collaboration, the perception of group dynamics, problem solving, decision-making, self-awareness, and moral commitment. This approach, combined with narrative pedagogy, gave tutors/mentors the opportunity to reflect on and practice the competences considered essential for the exercise of leadership, through interaction with their groups [53]. The tutors/mentors had the opportunity to shape the culture of the groups (by the way they welcomed them and interacted with them), to get involved in the process of change, to redirect them away from fear and towards positive possibilities, and to define strategies to persuade them to move forward [53]. This contributed to changing the perception that the tutors/mentors had of themselves, which strengthened their self-knowledge and, consequently, consolidated the construction of their identity as leaders [53].

- Realistic simulation:
  Hashish and Bajbeir [66] and Spigelmyer and Loughran [54] highlighted simulation as an effective pedagogical strategy for developing leadership in undergraduate nursing students.
  Hashish and Bajbeir [66] concluded that this practice gave students the opportunity to get involved, experiment, and experience realistic clinical contexts in safe and controlled environments, which favoured cognitive and affective learning, while at the same time boosting self-confidence, self-efficacy, and communication skills, which are considered essential aspects of effective leadership. It also promoted experiential learning teamwork and group dynamics and encouraged reflective practice.
  Similarly, Spigelmyer and Loughran [54] corroborated these conclusions, showing in their study that the use of this methodology supported the learning process and that it was enhanced by preparatory activities, namely orientation to readings and thematic reviews on conflict resolution, prioritisation, and communication techniques. In this study, the combination with interpretive pedagogy was shown to improve students’ ability to think critically about their interventions, enabling them to be able to adjust their decisions in real contexts and in different clinical practice scenarios [54].
  In general, the practice of realistic simulation was evaluated by the participants as positive, motivating, necessary, and promoting the development of leadership skills and was widely recognised by them for its proven benefits in their development [54,66]. In addition, it was also considered a cost-effective strategy for providing students with clinical experience, especially when it is not possible to carry it out in healthcare systems [54]. The teacher was once again seen as a facilitator of the whole process, considered responsible for creating an environment conducive to learning and encouraging proactive action on the part of the students [54,66].

- Flipped classroom:
  The study by Öz and Abaan [64] showed that the implementation of the flipped classroom methodology was associated with significant benefits in terms of leadership development. This is a methodology that inverts the traditional teaching dynamic from being teacher-centred to being student-centred [75]. In Öz and Abaan [64], students were asked to study the syllabus using videos, guided readings, and/or other teaching materials, including learning exercises, which were corrected and assessed by the teachers, who then gave feedback via an online teaching platform. The results obtained from this intervention showed that the students who took this innovative approach achieved better results than those who followed traditional teaching methodologies [64]. In this methodology, students had continuous access to course materials via an online platform, which allowed them to study flexibly and at their own pace [64]. This autonomy in the learning process not only fostered individual responsibility but also promoted greater involvement in the face-to-face sessions [64]. As a result, the students became more proactive and self-confident, characteristics considered fundamental to the development and exercise of leadership [64].
  The positive aspects associated with this methodology included flexibility in accessing content and prior preparation for classes: factors that were considered to have stimulated a sense of responsibility and initiative [64]. On the other hand, technical problems, the need for autonomous and individualised study, a high number of proposed activities, and the need for active participation were identified as being negative [64].

- Student-led conferences:
  The study by Pardo et al. [27] demonstrated the implementation of a student-led conference as a strategy to foster leadership development. In the analysis of the data collected through the application of the ES-SALI scale, Pardo et al. [27] found a significant increase in the total score obtained in the post-conference evaluation compared to the previous evaluation, especially in the “Impact and Influence” dimension, which indicated an improvement in the students’ perception of their leadership behaviours. This result suggests that practical experience as leaders, associated with organising an event and making strategic decisions, helped to strengthen self-confidence and the ability to influence processes and people, aspects that are considered essential in the training of competent leaders [27]. In addition, the active participation of the students, who were divided into two groups with different responsibilities (scientific committee and steering committee), was shown to develop collaborative and management skills, and the teacher’s support ensured the necessary supervision for the success of the whole event [27].
  Also relevant was the high level of satisfaction expressed by the different participants in the post-conference evaluation survey (which included students, lecturers, guest speakers, and health professionals) [27]. These data reinforce the effectiveness of the methodology used, demonstrating that practical involvement in leadership, when supervised and combined with reflective practice, can be a significant way of training future leaders in nursing [27].

- Student-run teams:
  Student-managed teams appear in a study by Reime et al. [71] as a strategy for developing leadership. In the study, it was shown that this strategy allowed students to take responsibility for managing groups of patients and colleagues under the supervision of experienced nurses, which promoted the reinforcement of competence from a theoretical and practical point of view and the development of essential skills, such as conflict resolution, decision-making, effective communication, interpersonal relationships, and work management [71].
  From analysing the students’ reflections on the experience, we can highlight the fact that they felt that their leadership skills were developed in an authentic and safe clinical environment, which enabled them to experience the challenges of professional practice in a real context [71]. The allocation of responsibilities, the continuous support of the supervising nurses, and the real challenges they faced (such as delegating tasks and organising care) were recognised as fundamental throughout the process [71].

- Camp-style leadership education programme:
  In the study by Oh and Lim [67], the Camp-Style Leadership Education Programme (CLEP) was developed with the aim of reinforcing leadership skills through an extracurricular approach. This programme was structured on the basis of a previous literature review, which resulted in the formulation of eight modules, focused on 13 concepts considered fundamental: (1) the concept of leadership; (2) leadership development; (3) self-knowledge and personal exploration; (4) self-esteem; (5) strategic vision and goal setting; (6) negotiation and conflict resolution; (7) stress management; (8) effective communication; (9) time management; (10) prioritisation; (11) self-management; (12) problem solving; and (13) interpersonal/human relations [67]. The methodology used was performance-centred, integrating different teaching strategies (lectures, individual and team activities, group discussions, team presentations, and teacher feedback), with the aim of giving students the opportunity to understand the need for and importance of leadership in the context of nursing practice [67].
  The analysis of the results showed that the group of students who actively participated in CLEP made significant gains in the areas of self-leadership, transformational leadership, and servant leadership, compared to the group who did not participate, thus confirming the programme’s effectiveness [67]. For this reason, the authors suggested integrating CLEP as part of the extracurricular training of undergraduate students, as well as expanding the programme to clinical nurses, with the aim of promoting continuous improvement in leadership skills [67].

- Dedicated education unit (DEU):
  The study by Pardo et al. [59] demonstrated the success achieved in developing leadership because of implementing a DEU in the context of an internship in a health centre. Their findings showed that the students assigned to this methodology registered a more significant increase in the perception of leadership behaviours (assessed through the application of the ES-SALI) at the end of the internship than the control group, especially in terms of the “Impact and Influence” and “Teamwork Skills” dimensions [59]. These students had the opportunity to take the lead in designing and implementing health education activities independently, both individually and in groups, under the supervision of a nurse, which undeniably contributed to their development as leaders [59]. In addition, both students and service users reported high levels of satisfaction, which reinforced the effectiveness of this intervention in the context of training future nursing professionals [59].
  These positive results, together with the high acceptability and potential for adaptation and transferability of the DEU model, have led the authors to suggest its expansion to different educational and socio-cultural contexts, to provide significant benefits for the development of leadership competences among nursing students [59].

This analysis has shown that each teaching–learning methodology contributes unique and valuable elements to the development of leadership in undergraduate nursing students. In addition, it was also found that combining and integrating these methodologies is an advantageous and innovative approach to undergraduate training, as it allows the specific benefits of different methods to be interlinked, maximising students’ learning potential and promoting a more enriching and meaningful educational experience. This is corroborated by Hashish and Bajbeir [66] and Spigelmyer and Loughran [54], who explored the combination of realistic simulation with interpretive pedagogies, and by Bright [53], who highlighted the advantage of combining tutoring/mentoring with narrative pedagogies.

Reflective practice is associated with all the methods and approaches presented, and its continuous and transversal application is advocated [53,54,66,67,71]. Through it, students consolidate the learning they have acquired and inter-relate theory with practice, transforming experiences into knowledge [76]. Reflecting on actions, decisions, and experiences not only improves understanding but also stimulates self-regulation and the development of metacognitive competences [76,77].

The teacher is seen as a facilitator of learning [27,54,57,64,66,67,74]. It is up to them to identify individual and collective student needs, adjust strategies, and create safe and motivating environments in which students can explore, make mistakes, reflect, and grow [74]. By facilitating debates, proposing challenges, and encouraging reflective practice, the teacher supports and guides the students in their development, ensuring the necessary opportunities for this [74].

It is, therefore, considered that the implementation of innovative methods and approaches to promote leadership development in nursing students is essential to ensure the training of effective leaders and, consequently, the provision of quality nursing care. However, for this development to be proven, it is essential that its impact is effectively measured. The rigorous evaluation of leadership development must be carried out using robust data collection instruments that make it possible to measure the changes and progress made in this area, because only through detailed and well-founded analyses will it be possible to adjust curricula and continually improve teaching methodologies. For this reason, the following section will be dedicated to presenting the data collection instruments used in the articles included.

### 4.3. Assessment of Leadership Development: Instruments and Data Collection Methods

Evaluating leadership development in undergraduate nursing students is an essential component for understanding the effectiveness of the teaching methodologies and educational models adopted. However, for this assessment to be rigorous and robust, it is essential to use specific tools that are properly structured and validated for the training context, to guarantee the objectivity, consistency, and validity of the results obtained. This will allow comparative analyses to be carried out, which will guide and support the implementation of changes and improvements in pedagogical interventions.

Analysing the articles included made it possible to identify various measuring instruments, which stood out for their specificity and relevance in the context of undergraduate training:

- Linares et al. [29] present in their study the translation, cross-cultural adaptation, and validation of the Self-Assessment Leadership Instrument (SALI) scale for the Spanish cultural context, giving rise to the ES-SALI. This scale consists of 40 items and was originally developed in 1988 by Smola [78,79], having been designed specifically to measure the leadership behaviours of nurses and nursing students. The Spanish version by Linares et al. [29] maintains the 40 items of the original scale, distributed in four dimensions: (1) strategic thinking; (2) emotional intelligence; (3) impact and influence; and (4) teamwork skills [27,29,59,72]. As a self-assessment scale, it has been proven that its application stimulates reflection and actively contributes to the construction of a leader’s identity, which is why it has been widely used in different studies and in different cultural contexts and can also be found in the studies by Baron et al. [72], Pardo et al. [27], and Pardo et al. [59];
- Karaman et al. [65] present the development of the Educational Leadership Scale for Nursing Students. This scale aims to assess educational leadership tendencies in nursing students and is made up of nine items, arranged in three dimensions: (1) scientific leadership; (2) Instructional leadership; and (3) Visionary leadership [65]. According to Karaman et al. [65], the application of this scale results in proven and extremely important benefits, since it will allow us to identify, develop, and consolidate educational leadership in nursing students over time, promoting its mastery [65];
- Hsieh et al. [70] used the Nursing Leadership Competence Assessment Scale for Undergraduate Nursing Students (NLCAS/UNS). This scale consists of 14 items and was developed by the authors with the aim of assessing leadership competence among nursing students following the implementation of an innovative curricular programme [70]. Its use allowed the authors to analyse the effectiveness of the leadership objectives developed and integrated into the curriculum, which allowed them to adjust them and assess their impact [70];
- Putra et al. [68], on the other hand, used the Pengembangan Perilaku Kepemimpinan Mahasiswa/Student Leadership Behaviour Development (PPKM/SLBD). This instrument consists of 13 items and was used to measure leadership development in nursing students after the restructuring of the curriculum programme with active learning methodologies, called the competency-based curriculum [68];
- Oh and Lim [67] used three scales to identify the effects of CLEP on leadership development: the 23-item Self-Leadership Questionnaire, developed by Prussia et al. [80] and modified by Lee [81]; the 12-item Multifactor Leadership Questionnaire, developed by Avolio and Bass [82] and modified by Sung [83]; and the 10-item Servant Leadership Instrument, developed by Greenleaf and Spears [84] and modified by Jun [85];
- Hashish and Bajbeir [66] applied Schwarzer and Jerusalem’s General Self-Efficacy Scale (GSES) [86], combined with a questionnaire developed by the researchers, with the aim of assessing the impact of management and leadership simulation on student development. Understanding students’ levels of self-efficacy was considered by the authors to be a crucial factor in leadership development, as it strengthens the construction of a leader’s identity and promotes the development of essential competences [66]. In this sense, self-efficacy emerges as a fundamental pillar, since it directly influences students’ confidence and ability to face challenges, make decisions, and mobilise resources to achieve the desired results [66];
- Sarnkhaowkhom et al. [69] explored the concept of entrepreneurial leadership in the context of undergraduate training, using a questionnaire developed by themselves: The Entrepreneurial Leadership of Nursing Student Questionnaire. This instrument consists of 36 items, organised into four dimensions: personal competency; management competency; proactive competency; and Technological competency [69]. According to the authors, entrepreneurial leadership not only fosters innovation and adaptability, but also prepares future nurses to deal with the current challenges of increasingly diverse healthcare contexts [69]. The incorporation of entrepreneurial leadership practices into undergraduate training is, therefore, advocated by Sarnkhaowkhom et al. [69], who consider it fundamental for training competent and resilient leaders capable of transforming nursing and promoting substantial improvements in the quality of the care provided;
- Finally, Spigelmyer and Loughran [54] used a 15-item tool from the TeamSTTEPS Learning Benchmarks to assess the impact of simulation on the development of leadership in students. The results obtained proved the effectiveness of the methodology applied and provided information on communication skills, teamwork, and identifying risks to patient safety, which, in turn, also encouraged critical reflection and personal development [54].

That said, we would emphasise the use of assessment tools as essential to guaranteeing the effectiveness of the pedagogical methodologies and educational models adopted. The diversity of instruments that resulted from analysing the articles showed a wealth of approaches and different dimensions that could be measured, since each tool offered a unique perspective and a detailed assessment of leadership development.

We therefore conclude that integrating these tools into the undergraduate training process has a significant impact on the development of this type of competence, since they promote more dynamic and reflective learning environments (due to the feedback they provide). In addition, we believe they will also make it possible to personalise and individualise teaching interventions, helping to identify areas for improvement, adapt strategies, and promote continuous development. All this justifies their great relevance, making them crucial in the preparation of future nurses, enabling them to deal with the complex and demanding challenges of today’s healthcare.

### 4.4. Conceptual Models and Curricular Programmes: Foundations for Leadership Development

The development of leadership in undergraduate training requires solid foundations which, based on conceptual models, guide the construction of effective curricular programmes [1,55,60,61,62,68,70].

Within the scope of this scoping review, innovative conceptual models and transformative curricular programmes were identified that have been shown to contribute significantly to the development of leadership in undergraduate nursing students.

One of the models identified was that of Miles and Scott [1]—The Leadership Development Model. It is a model structured around three fundamental dimensions, which promote the comprehensive development of leadership as a core competence in nursing practice: (1) knowing; (2) doing; and (3) being [1]. The “knowing” dimension refers to the domain of theoretical knowledge about leadership, while the “doing” dimension focuses on the development of practical competences for leading [1]. These two dimensions are operationalised in nine leadership competences/tasks, which provide a solid basis for students to understand and apply the following: (1) define objectives; (2) adjust values; (3) motivate; (4) manage; (5) achieve a viable level of unity; (6) explain; (7) serve as a symbol; (8) represent the group; and (9) renew [1]. The “being” dimension focuses on the leader’s perception of him/herself, and the authors point out that the “possession of the requisite skills to lead is of no value if an individual does not perceive leadership as a critical dimension of nursing practice or if the individual does not label themself a leader” [1]. This dimension is underpinned by seven critical values, which in turn guide the individual perceptions that must be developed to lead: (1) self-awareness; (2) congruence; (3) commitment; (4) collaboration; (5) common purpose; (6) controversy with civility; and (7) citizenship [1]. This approach is particularly important because it is recognised that effective leadership is not only about acquiring skills, but also about building an identity as a leader [1].

Context appears as the fourth dimension of the model, and the authors recognise that understanding it is essential to ensure effective leadership [1]. Understanding the environment and socio-cultural, organisational, and political dynamics (referred to as contextual intelligence) is essential for future nurses to adjust their leadership approach to the specific circumstances of each situation, responding in the most appropriate and adequate way to the needs of each context [1].

Miles and Scott [1] therefore advocate the inclusion of this model from the start of undergraduate training, emphasising its usefulness in designing teaching approaches that consider the individual characteristics of students and reinforce the perception that leadership is an intrinsic component of nursing. This will promote the development of the specific competences associated with each dimension, preparing students for leadership—the learning objective [1].

Hsieh et al. [70], on the other hand, present the development of an innovative educational framework, structured by objectives, designed to improve students’ knowledge and attitudes towards leadership in nursing. The study was conducted in three distinct phases and underpinned by rigorous methodological foundations.

In the first phase, the authors defined the learning objectives using the Delphi technique, which involved a panel of experts [70]. This method made it possible to reach a consensus on the essential leadership competences that should be fostered in students [70].

In the second phase, the defined objectives were integrated into the curricular programme and distributed strategically throughout the different curricular units of undergraduate training [70]. An overall learning objective was established (“demonstrating leadership competence in patient care and teamwork”) and three distinct levels, each with a different focus and with four sub-objectives: level 1, “recognizing achievements among leaders”; level 2, “experiencing personal characteristics, leading people, resource management, and vision building among nursing leaders”; and level 3, “applying leadership competence in patient care and teamwork” [70].

In the third phase, the effectiveness of the programme was evaluated by applying the NLCAS/UNS [70]. The results showed that the implementation of these objectives was effective, with significant improvements in the leadership competences of nursing students observed in different curricular years [70]. These findings emphasise the positive impact that a structured and progressive approach has on student development, as well as the importance of integrating coherent methodological practices adjusted to each objective [70].

In this vein, the studies by Putra et al. [68] and Stubin et al. [55] provide important evidence on the effectiveness of student-centred curricula focused on the progressive and practical development of leadership. Both studies highlight the importance of using innovative teaching strategies which, as in the structure proposed by Hsieh et al. [70], promote the integration of theoretical knowledge and practical skills, as well as critical reflection, preparing students for the real challenges of clinical and organisational practice.

Putra et al. [68] demonstrated that the implementation of a competency-based curriculum (CBC) was an effective approach to leadership development, as it favoured the development of critical thinking, problem-solving skills, a sense of responsibility, public speaking, and respect for others. In addition, they also proved that the introduction of active learning methodologies is a determining factor in improving leadership behaviours, with promising results in this context, placing the leadership behaviours of students integrated into the CBC at a higher average level [68]. With this, the authors have shown that CBC not only strengthens technical and interpersonal skills but also fosters students’ ability to take on leadership roles in the future, and that the focus on practical and interactive activities promotes a dynamic and engaging learning environment [68].

Stubin et al. [55], in turn, corroborates the previous findings of Putra et al. [68], highlighting the importance of implementing innovative teaching strategies in competency-based curricula (as advocated in the book *Essentials Core Competencies for Professional Nursing Education*, from the American Association of Colleges of Nursing), considering this combination essential for the training of future nurses. This study arose from an initiative by the American Association of Colleges of Nursing, which involved integrating new teaching approaches into the curriculum, focused on promoting the development of leadership, resilience, and self-care/well-being in nursing students [55]. The authors implemented 13 teaching and evaluation strategies, which were carefully planned and aligned with each other, highlighting their diversity and practical applicability: (1) 4Ms framework from the Institute for Healthcare Improvement, with the inclusion of a 5th M; (2) case study; (3) wellness wheel; (4) therapy dog interaction; (5) gratitude jar; (6) yoga; (7) building personal resilience; (8) stress first aid course; (9) video “why is psychological safety so important in healthcare?”; (10) alumni panel; (11) teacher-developed clinical reflection tool; (12) leadership expert speaker; and (13) charger nurse experience [55]. Thus, strategies such as the 5Ms framework, the case study, and the wellness wheel promote holistic learning, integrating technical and emotional competences [55]. These initiatives emphasise the importance of self-reflection and introspection, which are considered fundamental for students to develop resilience and self-care, preparing them for the emotional challenges of clinical practice [55]. On the other hand, innovative activities, such as therapy dog interaction, the gratitude jar, and yoga sessions, stand out for providing moments of stress relief and promoting students’ mental and physical well-being [55]. Initiatives more centred on leadership, such as the alumni panel and the charger nurse experience, facilitate students’ transition to professional practice, providing direct contact with the challenges and skills required to exercise leadership in nursing [55]. These strategies were implemented in a group of students during one semester, who expressed high levels of satisfaction with the teaching strategies adopted, providing positive comments and structured reflections [55]. Stubin et al. [55] recommend integrating these strategies from the first levels of the curriculum programme, ensuring progressive and consolidated learning. Furthermore, the sustainability of the initiatives, supported by external collaborations and reduced costs, makes this programme viable and replicable, offering a valuable framework for training resilient and effective nurses who are prepared to exercise effective leadership in the contexts in which they will work in the future [55].

Having reached this point, it is now important to focus on the concept of clinical leadership, which plays a central role in nursing practice. This concept was further developed by Jack et al. [62], who, through their concept analysis, identified the different factors that influence it: (1) interpersonal skills; (2) mastery of up-to-date clinical knowledge; courage; (3) confidence; (4) the ability to change and adapt; (5) teamwork skills; and (5) the ability to set an example. Based on this analysis, Jack et al. [62] proposed an operational definition of clinical leadership: “Nursing student clinical leadership is the application of theory and practice derived knowledge and skills demonstrating competence in interpersonal communication, having contemporary, evidence-based, clinical knowledge and being a role model from the outset of their exposure to the practice environment” (p. 5). This once again reinforces the need to integrate theory and practice [58,61,62,73] from the outset of training and clinical experience, recognising leadership as a complex competence that involves not only technical skills, but also personal characteristics, such as confidence and courage [62].

However, for students to effectively develop these skills, it is essential that educators play an active role in finding, creating, and prioritising opportunities that encourage and enable the exercise of leadership. The inclusion of activities in safe environments, where students can practice and improve their skills in a progressive and structured way, is considered by Jack et al. [62] to be a key point and is, therefore, strongly recommended by these authors. The systematic incorporation of these opportunities into the curriculum contributes significantly to the effectiveness of the development of clinical leadership in nursing, enabling future professionals to face the complex challenges of clinical practice with confidence and competence [62].

In the field of clinical leadership, Brown, Dewing, et al. [60] propose a conceptual model for its development, which emphasises the importance of integrating leadership in a structured way throughout training, according to a sequential and clear pedagogical approach, like that advocated by Hsieh et al. [70]. According to these authors, leadership development should be understood as a continuous process that begins with clarifying and recognising leadership responsibilities, progressively evolving towards empowering and emancipating students [60]. To achieve this goal, Brown, Dewing, et al. [60] suggest the use of transformative pedagogical strategies, such as tutorials, practical simulations, and directed learning activities, as they believe they allow students to experience leadership in real contexts, promoting deeper and more reflective learning. In addition, this model also emphasises the importance of understanding how students perceive their own leadership development, as this will provide valuable insights into the effectiveness of the teaching methodologies adopted [60]. In this sense, the use of self-assessment scales is highlighted as a fundamental tool for students to monitor their own progress, while at the same time enabling a continuous evaluation of the curriculum.

To complement this model, the same authors carried out another study with the aim of analysing the opinions of health professionals on the curricular content needed to train nursing students in clinical leadership [61]. The analysis revealed a significant consensus on the need to integrate content in a structured way throughout the curriculum, relating to three main dimensions: (1) knowledge, with themes identified relating to the role of the nurse, health and safety at work, risk management, and cultural diversity (among others); (2) skills, with the indication of the importance of developing written and oral communication skills, as well as the ability to deal with change, establish therapeutic relationships, and resolve conflicts (among others); and (3) behaviours, which include responsibility, assertiveness, honesty, respect, trust, commitment, and the ability to influence and set an example (among others) [61]. According to Brown, Crookes, et al. [61], this consensus strengthens the premise that clinical leadership should occupy a central position in undergraduate training programmes, which is justified by its proven direct impact on the training of competent future nurses who are capable of facing the current demands of healthcare.

We therefore conclude that the results obtained provide valuable guidelines for the development of more effective curricula aligned with leadership development needs, highlighting the importance of integrating theory and practice, ensuring continuous and structured development throughout the curriculum, and adopting innovative, student-centred teaching methodologies.

## 5. Limitations

This scoping review has made significant contributions to understanding leadership development in undergraduate nursing students, identifying valuable findings that underpin practice and research in the area. Even so, we recognise that the results obtained may not cover all the effective strategies available, nor fully reflect the perceptions, evaluation methods, and conceptual models and curricula related to the subject. In this sense, we consider it pertinent to carry out new, more-specific literature reviews that explore each of these dimensions in depth, contributing to building a more solid knowledge base and strengthening scientific evidence in the field of nursing leadership.

Another limitation identified refers to the fact that most of the studies included were conducted in countries with different financial and cultural contexts and educational systems, which may limit the transferability of the results to other regions, particularly contexts with fewer resources or less developed educational and health systems. This diversity of settings restricts the universal applicability of the conclusions, and it is necessary to recognise that cultural and economic factors have a direct influence on the adoption and effectiveness of the strategies used for leadership development.

Another relevant aspect to emphasise is the lack of studies evaluating the long-term impact of the leadership skills developed during undergraduate training. Although extensive immediate benefits have been identified, we believe it would also have been interesting to include how these competences impact on the development of future professional careers.

It should also be noted that the study focused on languages with the greatest presence in the international scientific literature—namely, Portuguese, Spanish, and English. Other languages were excluded due to the linguistic proficiency of the reviewers, which may constitute a limitation of the study.

## 6. Implications

The significant findings of this scoping review have several implications for the provision of care, nursing management, research, and teaching practice.

The implications for the provision of care are closely related to the proven benefits that effective leadership has for the NPE and for the quality and safety of the care provided [4,5,6]. Some studies have shown that better levels of job satisfaction, productivity, staff retention, the quality of care provided, the satisfaction of those receiving care, and adverse effects (with lower rates of mortality and nosocomial infections) are associated with effective leadership styles [4,5,6,7], which is why it is important to promote them from undergraduate training onwards.

For nursing management, the implications are equally substantial, as we believe that nurse managers will be able to use these findings to improve their practices and even adapt strategies that promote the assessment and development of leadership within their team.

As far as research is concerned, we believe that the results obtained also have substantial implications for the advancement of scientific knowledge in leadership, providing solid foundations for the development of new studies. Therefore, in addition to the suggestions made above because of the limitations (namely more-specific literature reviews), we recognise that it would be important to carry out longitudinal studies focused on the real and long-term impacts of leadership development in undergraduate training, as well as its influence on the transition to professional practice. It would also be interesting to (1) explore the adaptation and implementation of innovative teaching methods that are adaptable to different cultural and economic contexts; (2) analyse the possibility and effectiveness of integrating new technologies (such as virtual reality) in teaching and promoting leadership; and (3) evaluate the impact of using interdisciplinary approaches in this area, as we believe that collaboration between students from different areas of health could enrich the understanding and practice of leadership in real contexts.

The implications for teaching practice stand out as highly relevant, since the results obtained indicate the need to restructure curricula so that the teaching of leadership is transversal, continuous, progressive, consistent, sustained, and articulated, incorporating different disciplines in a holistic way [1,27,29,38,53,54,55,56,57,58,59,60,61,62,63,64,65,66,67,68,69,70,71,72,73]. It should, therefore, not be a one-off training event, but structured and integrated throughout undergraduate training.

## 7. Conclusions

This scoping review unequivocally reveals the relevance of leadership development in undergraduate training, highlighting it as a crucial dimension for nursing practice. The analysis of the 25 articles included made it possible to identify four thematic categories, the analysis of which allowed for a more in-depth understanding of how leadership development is characterised in the undergraduate training of nursing students—students’ perceptions of leadership, strategies to train leaders in nursing, the evaluation of leadership development, and conceptual models and curricula.

In general, the students recognised leadership as an indispensable skill for nursing practice, with a direct impact on the quality and safety of the care provided. However, they also expressed a general perception of insufficient preparation to take on a leadership role, highlighting an urgent need to restructure curricula so that they integrate its teaching from the initial years of training.

On the other hand, various strategies were also identified to promote the development of leadership, and student-centred pedagogical approaches were highlighted, with the teacher taking on the role of a learning facilitator.

Different leadership assessment tools were also identified, which allow progress to be monitored and promote the development and consolidation of an identity as a leader.

Finally, the conceptual models suggest that the integration of leadership teaching and development should be done in a structured, continuous, and transversal way, recognising its importance not only as a competence but also as a value.

## Figures and Tables

**Table 1:** Summary of the contribution of the articles included to the thematic categories identified.

Selected Articles		Thematic Categories			
		Students’ Perception of Leadership	Strategies To Training Leaders in Nursing	The Evaluation of Leadership Development	Conceptual Models and Curricular Programmes
1	Aydogdu, A. (2023) [38]	•			
2	Baron, et al. (2024) [72]	•		•	
3	Bright, A. (2019) [53]		•		
4	Brown, A., Dewing, J. & Crookes, P. (2016) [60]				•
5	Brown, A., Crookes, P. & Dewing, J. (2016) [61]				•
6	Démeh, W. & Rosengren, K. (2015) [73]	•			
7	dos Santos, I. et al. (2021) [56]	•	•		
8	Hashish, E. & Bajbeir, E. (2022) [66]		•	•	
9	Hsieh, L. et al. (2022) [70]			•	•
10	Jack, K. et al. (2022) [62]				•
11	James, A. et al. (2022) [63]	•			
12	Karaman, F. et al. (2023) [65]			•	
13	Linares, P. et al. (2020) [29]			•	
14	Melo, G. et al. (2020) [57]	•			
15	Miles, J. & Scott, E. (2019) [1]				•
16	Oh, S & Lim, J. (2019) [67]		•	•	
17	Öz, G & Abaan, S. (2021) [64]		•		
18	Paim, C. et al. (2021) [58]	•			
19	Pardo, M. et al. (2021) [27]		•	•	
20	Pardo, M. et al. (2022) [59]		•	•	
21	Putra, A. et al. (2021) [68]		•	•	•
22	Reime, M. et al. (2022) [71]	•			
23	Sarnkhaowkhom, C. et al. (2022) [69]			•	
24	Spigelmyer, P. & Loughran, M. (2022) [54]		•	•	
25	Stubin, C. et al. (2024) [55]				•

Legend: “•” indicates that the study contributed to the thematic category

## Data Availability

For the data that support the results presented, please contact the authors of this review.

## Supplementary Materials

The following supporting information can be downloaded at https://www.mdpi.com/article/10.3390/nursrep15050160/s1. Supplementary File S1: detailed search strategy. Supplementary File S2: data extraction table. Supplementary File S3: synthesis by meta-aggregation.

## Public Involvement Statement

There was no public involvement in any aspect of this research.

## Guidelines and Standards Statement

This manuscript was drafted against the Preferred Reporting Items for Systematic Reviews and Meta-Analyses Extension for Scoping Reviews (PRISMA-ScR) [48].
