# Supplementary material for: Leadership Development in Undergraduate Nursing Students: A Scoping Review
Source: Nurs Rep. 2025 May 2;15(5):160. doi: 10.3390/nursrep15050160 (PMC12114338; doi:10.3390/nursrep15050160)
Supplement: Supplementary file 1 [file nursrep-15-00160-s001.zip › Supplementary File S1_Detailed search strategy.pdf]

Supplementary File S1: Detailed search strategy

	P (Population)<br>Undergraduate nursing students	C (Concept)<br>Leadership development	C (Context)<br>Nursing education
**Natural language terms**	- Nursing students<br>- Nurse student<br>- Baccalaureate student/s<br>- Undergraduate student/s	- Leadership<br>- Leadership development<br>- Lead<br>- Leading<br>- Leader	- Nursing education
**MeSH**	- Students, Nursing	- Leadership	- Education, Nursing, Baccalaureate<br>- Nursing Education
**CINAHL Headings**	- Students, Nursing<br>- Students, Nursing, Practical	- Leaders<br>- Leadership	- Nursing Education<br>- Nursing Education, Practical
**Thesaurus**	- Nursing Students	- Leaders<br>- Leadership<br>- Leadership training	- Nursing Education

Keyword-inclusive strings were thoroughly searched within the title, abstract, and keyword sections across the databases.

CINAHL Ultimate

Interface: EBSCOhost Research Databases.
Search Screen: Advanced Search.
Final Search Date: 25/10/2024
Search expressions:
S1: Nurs* student* OR Students, Nursing OR Baccalaureate Student* OR Undergraduate Student* OR Students, Nursing, Practical → **22,779**
S2: Leadership OR Leadership development OR Leadership training OR Lead* → **65,871**
S3: Nursing Education OR Education, Nursing, Baccalaureate OR Nursing Education, Practical → **10,333**
S4 → **S1** AND **S2** AND **S3** → **8**
Of the available limitations, the ones we used were: Publication date from 2014 to 2024 → **5**

MEDLINE Ultimate

Interface: EBSCOhost Research Databases.
Search Screen: Advanced Search.
Final Search Date: 25/10/2024
Search expressions:
S1: Nurs* student* OR Students, Nursing OR Baccalaureate Student* OR Undergraduate Student* OR Students, Nursing, Practical → **20,822**
S2: Leadership OR Leadership development OR Leadership training OR Lead* → **148,567**
S3: Nursing Education OR Education, Nursing, Baccalaureate OR Nursing Education, Practical → **9,379**
S4 → **S1** AND **S2** AND **S3** → **4**
Of the available limitations, the ones we used were: Publication date from 2014 to 2024 → **2**

Scopus

Interface: Scopus.
Search Screen: Advanced Search.
Final Search Date: 25/10/2024
Search expressions:
("Nurs* student*" OR "Students, Nursing" OR "Baccalaureate Student*" OR "Undergraduate Student*" OR "Students, Nursing, Practical") AND ("Leadership" OR "Leadership development" OR "Leadership training" OR "Lead*") AND ("Nursing Education" OR "Education, Nursing, Baccalaureate" OR "Nursing Education, Practical")
Of the available limitations, the ones we used were: Publication date from 2014 to 2024, Language (English and Portuguese) and Open Access → **235**

Sciencedirect

Interface: ScienceDirect.
Search Screen: Advanced Search.
Final Search Date: 25/10/2024
Search expressions:
Wildcards '' are not supported, in accordance, terms were completed. Only 8 Boolean operators are allowed to use.*
("Nursing students" OR "Baccalaureate Students" OR "Undergraduate Students") AND ("Leadership" OR "Leadership development") AND ("Nursing Education" OR Nursing, Education, Practical")
Of the available limitations, the ones we used were: Publication date from 2014 to 2024, Subject areas (Nursing and Health professions), Language (English) and Open Access → **291**

Web of Science Core Collection

Interface: Web of Science.
Search Screen: Advanced Search.
Final Search Date: 25/10/2024
Search expressions:
("Nurs* student*" OR "Students, Nursing" OR "Baccalaureate Student*" OR "Undergraduate Student*" OR "Students, Nursing, Practical") AND ("Leadership" OR "Leadership development" OR "Leadership training" OR "Lead*") AND ("Nursing Education" OR "Education, Nursing, Baccalaureate" OR "Nursing Education, Practical")
Of the available limitations, the ones we used were: Publication date from 2014 to 2024, Language (English and Portuguese) and Open Access → **137**

LILACS

Interface: Virtual Health Library.
Search Screen: Advanced Search.
Final Search Date: 25/10/2024
Search expressions:
("Nurse student" OR "Nursing Students" OR "Students, Nursing" OR "Baccalaureate Student" OR "Baccalaureate Students" OR "Undergraduate Student" OR "Undergraduate Students" OR "Students, Nursing, Practical") AND ("Leadership" OR "Leadership development" OR "Leadership training" OR "Lead" OR "Leading" OR "Leader") AND ("Nursing Education" OR "Education, Nursing, Baccalaureate" OR "Nursing Education, Practical")
Of the available limitations, the ones we used were: Publication date from 2014 to 2024 and Language (English, Portuguese and Spanish) → **5**

RCAAP (Open Access Scientific Repository of Portugal)

Interface: RCAAP.
Search Screen: Advanced Search.
Final Search Date: 25/10/2024
Search expressions:
("Nurse student" OR "Nursing Students" OR "Students, Nursing" OR "Baccalaureate Student" OR "Baccalaureate Students" OR "Undergraduate Student" OR "Undergraduate Students" OR "Students, Nursing, Practical") AND ("Leadership" OR "Leadership development" OR "Leadership training" OR "Lead" OR "Leading" OR "Leader") AND ("Nursing Education" OR "Education, Nursing, Baccalaureate" OR "Nursing Education, Practical")
Of the available limitations, the ones we used were: Publication date from 2014 to 2024 → **2**
