# Supplementary material for: Leadership Development in Undergraduate Nursing Students: A Scoping Review
Source: Nurs Rep. 2025 May 2;15(5):160. doi: 10.3390/nursrep15050160 (PMC12114338; doi:10.3390/nursrep15050160)
Supplement: Supplementary file 1 [file nursrep-15-00160-s001.zip › Supplementary File S2_Data extraction table.pdf]

Supplementary File S2: Data extraction table

	Author(s)<br>Year of publication<br>Country	Title	Objectives	Study design	Participants	Context<br>Data collection	Relevant concept(s) of the review question<br>Data collection instrument(s)	Main results
**1**	Aydogdu, A.<br>2023<br>Brazil	Perceptions of nursing students about leadership: A qualitative study.	Identify the perceptions of undergraduate nursing students about leadership and provide recommendations for educating future nurses to exercise leadership.	Descriptive qualitative study.	30 undergraduate nursing students from different semesters of the course.	The study was carried out at universities in the south-east of Brazil and the data was collected in February 2023.	Leadership, development of leadership competences, students' perceptions and academic training.<br><br>A questionnaire was administered online, with open-ended questions about leadership.	The results were grouped into 3 themes:<br>• Opinions on leadership: The students considered leadership to be an essential skill for ensuring quality care, resolving complications, promoting teamwork, minimising errors and acting as a role model/example.<br>• Necessary competences of a nurse leader: 70% of students said they did not feel prepared to lead a nursing team in the future. They identified the need to develop technical, human (especially effective communication and the creation of working environments that motivate the team, resolve conflicts and lead to a common goal) and conceptual (to understand and analyse complex situations) skills in order to exercise effective leadership;<br>• Suggestions for educating nursing students in leadership: 40% of the participants said they had not yet had any lessons on leadership in nursing, despite the fact that the majority had already completed half of their course. Promoting leadership development from the start of training (renewing and updating the school curriculum), theoretical approaches orientated towards leadership (more specific classes and extracurricular activities such as courses, congresses and conferences), continuing training (in different semesters) and developing leadership through practice (simulated practice and role-playing) were mentioned as suggestions for the future.
**2**	Baron, et al.<br>2024<br>Israel and United Kingdom	Dimensions of clinical leadership among pre-registration nursing students: A cross-sectional study between two countries.	Explore pre-registration nursing students' self-perceptions of leadership behaviours in clinical practice and compare students' perceptions of leadership between two countries: Israel and the United Kingdom.	Cross-sectional survey design.	140 undergraduate nursing students from two universities (79 from a university in Israel and 61 from a university in England).	Study carried out during the COVID-19 pandemic at universities in two countries: Israel and England. The data was collected between October 2021 and April 2022.	Leadership, leadership behaviours, clinical leadership, development of leadership skills, students' perceptions and academic training.<br><br>An online survey was carried out with the translated Hebrew and English versions of the ES-SALI scale.	The participants generally considered themselves to be involved in leadership behaviours or to possess the attributes related to leadership behaviours, recognising their importance. However, they emphasised the need for more consistent application.<br>Curricula should be designed to include more opportunities for leadership development, with the aim of strengthening students' self-esteem, effectiveness and performance in terms of strategic thinking, emotional intelligence, impact and influence, and teamwork skills.<br>The results indicated significant differences between the two countries, with students from England obtaining higher marks than students from Israel. These differences can be attributed to variations in curricula (number of clinical placements carried out in different contexts; number of years of training), sociodemographic characteristics (age), previous professional experience and previous attendance at leadership courses. In addition, Israeli students in the later years of the course obtained higher scores in all dimensions than students in the early years.<br>Students with previous work experience and participation in leadership courses generally obtained higher scores.
**3**	Bright, A.<br>2019<br>United States of America (USA)	Practicing leadership skills though peer mentoring and teaching: The lived experience of BSN students.	To explore how the tutor/mentor experience contributes to the development of leadership competences among undergraduate nursing students.	Phenomenological study.	2 advanced nursing students (in their final year) 1 first-year student and 2 recent *alumni*/newly graduated nurses who were preparing for the RN licensing exam (NCLEX).	The study was carried out as part of a bachelor's degree programme in nursing at a private university in the United States and the data was collected through a focus group held at the end of the semester (the academic year was not specified).	Leadership, development of leadership competences, tutoring/mentoring and narrative pedagogy.<br><br>The data was collected through a focus group involving the tutors/mentors and two members of the teaching staff.	The results indicate that the tutors/mentors took advantage of the opportunities to practise and reflect on the competences of effective leadership.<br>Involving advanced students in mentoring, teaching and leading newer students, in combination with a narrative pedagogical approach, was a cost-effective and efficient way to reinforce learning, provide support for beginning students, develop leadership skills among advanced students and strengthen the programme in general.<br>The participants reported that this experience made them realise their own leadership skills and competences (self-knowledge), gave them a new perspective on teaching and leading and helped them feel more confident in their ability to influence and guide others. In addition, they also observed that their presence and support helped relieve the stress of the beginner students, providing them with a more familiar and welcoming environment during the most challenging times, such as exams.<br>Narrative pedagogy is seen as extremely useful for promoting the development of leadership competences and attributes in future nurses.<br>It is suggested that extracurricular activities be organised, such as mentoring and involving students in the teaching-learning process, so that they can act as leaders in real situations.
**4**	Brown, A., Dewing, J. & Crookes, P.<br>2016<br>Australia	Clinical leadership and pre-registration nursing programmes: A model for clinical leadership and prospective curriculum implementation and evaluation research strategy.	To present a conceptual model for the development of clinical leadership in undergraduate nursing programmes and a proposal for an implementation plan.	Study of the theoretical and analytical nature.	The research included nursing professionals (clinicians, managers and academics) in Australia, but the exact sample size was not specified.	Proposal for a conceptual model for the development of clinical leadership in undergraduate training programmes.<br>The period of the study was not specified.	Clinical leadership, conceptual model, curriculum-pedagogy *nexus*, curriculum development, evaluation and implementation.<br><br>A comprehensive analysis of theoretical and empirical literature from 1974-2015 was carried out with the aim of identifying and verifying the antecedents relating to clinical leadership in curricular content, as well as the most effective pedagogical approaches for its implementation in undergraduate nursing programmes.	The article presents a PhD project that proposes a conceptual model for the development of leadership in undergraduate nursing programmes, emphasising the need to integrate leadership into the curriculum in a structured, sequential and clear way.<br>The research identified leadership content in academic curricula and highlighted the importance of adopting a pedagogical approach that promotes the development of leadership skills among students. The authors point out that clinical leadership can be made explicit through transformative/emancipatory teaching and learning approaches and suggest integrating tutorials, practical simulations and/or directed learning activities in order to reflect the enlightenment-empowerment-emancipation *continuum*.<br>The next step is to carry out a focus group, collect the opinion of former participants (expert opinion) and apply a national online survey, with the aim of collecting data on the curricular content relevant to leadership in nursing.<br>It is also planned to develop a leadership self-assessment scale, which will be used to measure the effectiveness of the curriculum

								(of the proposed model) and the students' understanding of clinical leadership throughout the course.<br>It is suggested that learning opportunities be identified and that academics and clinical supervisors be involved in evaluating the curriculum.
**5**	Brown, A., Crookes, P. & Dewing, J.<br>2016<br>Australia	Clinical leadership development in a pre-registration nursing curriculum: What the profession has to say about it.	To identify the opinions of professionals on the indicative curricular contents proposed for the development of clinical leadership in a pre-registration nursing course in Australia.	Multi-method study/mixed methodology study.	416 nurses nationwide: clinicians (347), managers (163) and academics (44).	The study was carried out nationwide, using an online survey available for 8 weeks, without the use of reminders.	Leadership and management in nursing, clinical leadership, development of leadership knowledge, skills and behaviours, curriculum and academic training.<br><br>A questionnaire was developed, based on the literature review previously carried out. To ensure the validity of the questionnaire, experts in the field were asked for their opinion and a focus group was held. The questionnaire developed contained demographic questions and questions about the relevance and importance of the proposed curriculum content. It was distributed nationwide in an online format.	Clinical leadership was identified as an essential competence in nursing, the development of which should be supported in the pre-registration nursing programme, and it is up to those responsible for preparing students to influence its development.<br>A consensus was reached among the participants regarding the relevant and important leadership content in pre-registration nursing programmes.<br>The results obtained can guide the development of curricular programmes, as they have identified a set of curricular contents (approved by professional nurses) that could be part of the programme.<br>The survey can be used as an auditing tool.
**6**	Déméh, W. & Rosengren, K.<br>2015<br>Jordan and Sweden	The visualisation of clinical leadership in the content of nursing education: A qualitative study of nursing students' experiences.	Describe the clinical leadership experiences of nursing students during their final year of training	A descriptive qualitative study.	20 undergraduate nursing students in their final year of training, who completed a course called "Management and Leadership in Nursing-Clinical" at the University of Jordan.	The study was carried out at a university in Jordan and the data was collected between May and July 2014.	Clinical leadership, nursing management, development of leadership and management skills and academic training.<br><br>Twenty written narratives were collected, developed from two open questions relating to the students' experiences of clinical leadership.	The study revealed that the students considered clinical leadership to be a valuable tool in the transition from theory to practice. They emphasised its importance as a source of security for nurses, an eye-opener, a role model and a means of bridging the gap between theoretical academic training and practice. It was seen by the participants as an essential element that facilitates the integration of theoretical knowledge into practice, contributing to the provision of safe and effective care. In addition, support and collaboration between members of the healthcare team were highlighted as factors contributing to the development of self-confidence and autonomy in nursing practice.<br>The implications of this study for education and practice are significant. It highlighted, on the one hand, the importance of integrating clinical leadership into the academic curriculum in order to prepare students for today's complex healthcare environments and, on the other, the need for training to be practical and not just theoretical, arguing that this approach will allow students to develop management and leadership skills from the start of their training.<br>In addition, the importance of collaboration between educators and health institutions was also addressed, in order to guarantee a safe and effective transition for newly qualified nurses into professional practice and to promote an environment that values leadership and quality of care.
**7**	Dos Santos, I. et al.<br>2021<br>Brazil	Theorical-practical articulation of the continuous learning of leadership in nursing in light of Peter Senge.	To analyse the theoretical and practical articulation of the constant learning of leadership in undergraduate nursing courses.	Case study with a qualitative approach.	40 undergraduate students enrolled in the nursing programme.	The study was carried out at a federal public university in north-eastern Brazil and the data was collected between November 2017 and July 2018.	Leadership in nursing, development of leadership skills, critical reflection, theoretical-practical articulation and academic training.<br><br>The study used semi-structured interviews, workshops and documentary analysis to gather data.	The students recognised the importance of teaching leadership and identified elements that could contribute to its development. They suggest that the subject of leadership should be introduced in a cross-curricular way from the beginning of the course, articulating with different subjects. In addition, they propose implementing pilot projects, videos, workshops, forums and other events to talk about leadership. The importance of practical experiences and teachers' narratives were highlighted as valuable tools for developing leadership skills, emphasising that learning from real experiences can positively influence future decisions.<br>The study highlighted the need to reinforce educational practices throughout the teaching-learning process, right from the start of training, recommending the use of active methodologies and experiences related to leadership development.<br>More flexible curricula and better integration between theory and practice are suggested, as is the use of innovative approaches that encourage reflection. However, there is still room for improvement with a view to better integrating theory into practice.
**8**	Hashish, E. & Bajbeir, E.<br>2022<br>Saudi Arabia	The Effect of managerial and leadership training and simulation on senior nursing students'career planning and self-efficacy.	To determine how training and simulation in management and leadership affect the career planning knowledge, career choice and self-efficacy of senior nursing students.<br>To explore how students perceive the training they receive.	Mixed methodology study.	77 final-year nursing students from a nursing college in Saudi Arabia.	The study was carried out at the College of Nursing-Jeddah (COM-J), King Saud bin Abdul-Aziz University for Health Sciences, National Guard Health Affairs, Saudi Arabia. The intervention and data collection took place during the spring semester of 2021 (academic year 2020-2021).	Management and leadership, academic training and simulation in management and leadership.<br><br>The study was carried out in 3 stages: pre-test, intervention (which consisted of 12 programmed sessions with interactive simulations), post-test and data analysis.<br>For the quantitative data, a quasi-experimental design was used, utilising a pre-test-post-test to assess the effect of the intervention on students' knowledge of career planning, career choice and self-efficacy. A questionnaire (developed by the researchers on the basis of a previous literature review) and a scale (the Schwarzer and Jerusalem Self-Efficacy Scale 2013) were used to collect data before and after the intervention.<br>For the qualitative data, an exploratory approach was taken, asking students to answer two open-ended questions about their experience on the course and how it could be improved.	The results indicate that the management and leadership training made a significant contribution to the students' self-efficacy. After the training, there was a considerable improvement in their self-efficacy scores, which suggests that practising leadership and management skills during the sessions increased their confidence and communication skills.<br>From the analysis of the students' open-ended responses, 4 main important themes emerged that should be emphasised throughout the training: student involvement; experiential learning and reflective practice; teamwork and group dynamics; the role of the teacher as facilitator. The simulation sessions were highlighted by the students as positive, motivating, necessary and promoting the development of self-confidence.<br>The conclusions of this study emphasise an important impact at the pedagogical and clinical level, reinforcing the need to integrate and promote leadership education consistently throughout nursing training. This approach will enable students to develop the necessary leadership skills from the earliest stages of their training, preparing them for the challenges of today's healthcare contexts.
**9**	Hsieh, L. et al.<br>2022	Improving leadership competence among	Identify baccalaureate nursing leadership	Multi-method study, structured in 3 phases/mixed	12 experts (5 nurse managers and 7 academic	The study was carried out at a university in Taiwan and the	Leadership competences, development of leadership competences, curriculum integration,	The study resulted in the identification and development of learning objectives related to leadership, which were integrated into the nursing degree programme curriculum. This integration

	Taiwan	undergraduate nursing students: Innovative objetives deveolpment, implementation, and evaluation.	objectives and evaluate their effectiveness when integrated into a nursing degree curriculum.	methodology study.	experts), all with master's degrees and/or doctorates.	data was collected between August 2015 and July 2017.	practical training and involvement of nurse leaders.<br><br>In phase 1, the Delphi technique was carried out, involving a panel of clinical and academic experts, to identify the learning objectives related to baccalaureate nursing leadership competences.<br>In phase 2, the objectives were integrated into the curriculum.<br>Phase 3 assessed the effectiveness of the leadership objectives using a pre-test/post-test design. The Nursing Leadership Competence Assessment Scale for Undergraduate Nursing Students (NLCAS/UNS) was used for data collection.	resulted in a significant improvement in students' leadership skills over the course of the academic year.<br>Considering the complexity inherent in leadership, a single year may not be enough to bring about significant changes, so this process must be continuous and carried out throughout academic training.<br>The authors recommend implementing more effective practical training that allows students to fulfil leadership roles. In addition, they suggest, on the one hand, inviting nurses in leadership positions to share their knowledge in the field with students, and on the other hand, evaluating the perceptions of teaching staff on leadership in nursing and carrying out longitudinal monitoring of the results of graduates, serving as a reference for teaching leadership in nursing.
**10**	Jack, K. et al.<br>2022<br>United Kingdom	Clinical leadership in nursing students: A concept analysis.	Carry out a conceptual analysis of clinical leadership in nursing students.	Conceptual analysis.	The study had no direct participants, but was aimed at undergraduate nursing students and registered nurses who supervise students in clinical teaching.	Theoretical contributions regarding the concept of clinical leadership in undergraduate nursing students.<br>The length of the study was not specified.	Clinical leadership and leadership competences in nursing students.<br><br>A conceptual analysis was carried out using the 8-step process proposed by Walker and Avant (2014).	Analysing the concept revealed several factors that influence nursing students' clinical leadership: interpersonal skills, possession of up-to-date clinical knowledge, courage, confidence, capacity for change, teamwork skills and the ability to act as a role model for others.<br>Based on these conclusions, an operational definition was proposed: Clinical leadership for nursing students is the application of knowledge and skills derived from theory and practice, translated into competences that include effective interpersonal communication, mastery of up-to-date clinical knowledge based on scientific evidence, and the ability to act as a role model for others from the outset of their experience in the context of nursing practice.<br>Recommendations that emerge from this analysis include the need for educators to identify and prioritise opportunities for students to exercise leadership, at all levels, so that students practice and rehearse their skills in a safe environment.
**11**	James, A. et al.<br>2022<br>England	Perceptions and experiences of leadership in undergraduate nurse education: A narrative inquiry.	To gain an understanding of the perceptions and leadership experiences of final year nursing students, educators and senior nurses.<br>To explore the perceptions of final year nursing students, educators and senior nurses regarding their expectations in terms of leadership competences for nurses who join the register.<br>To explore the attitudes of students, educators and senior nurses towards the effectiveness of preparation through education for the role of leadership in nursing.	A qualitative, narrative study.	6 academics with varied experience, 5 final year nursing students and 1 senior nurse.	The study was carried out at a university in England and in a clinical setting and the data was collected at the beginning of 2020.	Leadership, development of leadership competences, students' perceptions and academic training.<br><br>Semi-structured interviews and photographic elicitation were used to collect data.<br>The interviews were carried out at the beginning of 2020, recorded and transcribed for later analysis, during which the participants were encouraged to narrate their experiences and perceptions of leadership. At the end of each interview, each participant was shown a set of photographic images of nature and then asked to freely associate their thoughts and emotions with leadership experiences.	The results of this study revealed that the perceived characteristics of leadership were strongly influenced by the participants' experiences and learning, reflecting their emotional perceptions and positive and negative attitudes. Leadership development was positioned between practical experience and theoretical knowledge, which emphasised the importance of a clear alignment throughout the students' training.<br>The results showed that the students' expectations and self-image did not always align with the image of effective leaders and that negative experiences led to worries and feelings of not being prepared for leadership.<br>The participants stressed the importance of a continuous process of leadership development, self-awareness and reflection, advocating the combination of theory and practice, through experience and the visualisation of leadership through models.<br>Self-awareness, emotional intelligence and reflection on learning and leadership development were particularly valued
**12**	Karaman, F. et al.<br>2023<br>Turkey	Development of the educational leadership scale for nursing students: A methodological study.	To develop the educational leadership scale for nursing students and examine its validity and reliability.	Methodological study.	280 nursing students enrolled in a bachelor's degree in nursing.	The study was carried out at a private nursing school in Istanbul (higher education) and the data was collected between February and April 2022.	Educational leadership in nursing, Bloom's taxonomy, scientific, instructional and visionary leadership and the development and validation of measurement instruments.<br><br>The data collection instrument used was The Educational Leadership Scale for Nursing Students.	The study highlights the importance of educational leadership in nursing, emphasising that nurses play a fundamental role as educational leaders, as they are responsible for teaching-learning activities aimed at groups in health and educational institutions. Educational leadership is seen as an essential competence for nursing practice, and is especially emphasised in nurses with roles of greater responsibility. It is therefore crucial that nurses learn, develop and consolidate it over time, on an ongoing basis.<br>As a result of this study, an educational leadership self-assessment scale was developed and validated, with the aim of evaluating educational leadership tendencies in nursing students. It was based on Bloom's Taxonomy and consists of 19 items, which are organised into 3 factors/dimensions: Scientific Leadership, Instructional Leadership and Visionary Leadership. Its use will make it possible to identify, develop and consolidate educational leadership in nursing students, promoting its mastery over time.<br>The validation of the scale showed stability in all dimensions, as evidenced by the absence of statistically significant differences between the test and retest results, as well as the strong or very strong positive correlation between the scores obtained.
**13**	Linares, P. et al.<br>2020<br>Spain	Dimentions of leadership in undergraduate nursing students. Validation of a tool.	To identify the leadership dimensions, present among nursing students in the Self-Assessment Leadership Instrument.	Methodological study.	280 students enrolled on the nursing degree course in the 2017-2018 academic year, from the first to the fourth-year.	The study was carried out at a university in Barcelona and the data was collected between October 2017 and May 2018.	Leadership, transformational leadership, development of leadership competences and translation and cultural adaptation of measurement instruments.<br><br>The research instruments used were the Self-Assessment Leadership Instrument, the General Self-Efficacy Scale (convergent scale) and the Lille	The translation and cultural adaptation of the Self-Assessment Leadership Instrument for the Spanish cultural context resulted in the identification of 4 leadership dimensions: (1) Strategic Thinking; (2) Emotional Intelligence; (3) Impact and Influence; and (4) Teamwork Skills.<br>These dimensions are considered essential leadership competences, the promotion and evaluation of which are indispensable in the context of training nursing students. This understanding may be essential for structuring more specific

							Apathy Rating Scale (divergent scale).	pedagogical interventions in undergraduate nursing programmes.<br>The study concluded that the Spanish version of the Self-Assessment Leadership Instrument is a valid and reliable instrument that makes it possible to assess the impact of educational initiatives and students' progress in terms of leadership development.
**14**	Melo, G. et al.<br>2020<br>Brazil	Nursing student's perception about leadership learning in hospital emergency situations	To understand nursing students' perceptions of learning to lead in hospital emergency situations.	Qualitative study.	15 undergraduate nursing students, enrolled in the subjects "Curricular Internship I and II, eighth and ninth periods of the course" (students in the final periods of the course).	The study was carried out at a public university in the south of the state of Minas Gerais, Brazil. The data was collected between November 2017 and April 2018.	Leadership in nursing, development of leadership competences, emergency situations and academic and practical training/internship.<br>A semi-structured interview was used, guided by a script made up of socio-demographic questions and four guiding questions relating to the student's perception of their leadership development process.	The students recognised the nurse's role as a leader in emergency situations, highlighting that learning this competence is related to opportunities to experience situations during clinical practice. However, they identified that their status as students limits their opportunities to experience emergency situations, which is recognised as a barrier to learning the role of leader in this context and which can generate feelings of fear and insecurity.<br>As facilitators, the study highlighted internships in Intensive Care Units (which allow greater contact with emergency situations), realistic simulations (which promote learning in a safe and controlled environment) and the qualified intervention of the teacher (who plays a fundamental role in serving as a point of reference for students, guiding them in building their autonomy, without confining them to a passive or merely expectant position).
**15**	Miles, J. & Scott, E.<br>2019<br>United States of America	A new leadership development model for nursing education.	Establish an integrated leadership development model for pre-licensure nursing students that recognises leadership as a fundamental competence for nursing practice and promotes and develops scholarships for teaching leadership in nursing.	Study of the theoretical and analytical nature.	The study had no direct participants, but was aimed at undergraduate nursing students, as well as all those involved in their training (educators and nursing professionals).	Relative theoretical contributions that led to the development of a leadership development model in nursing. The period of the study was not specified.	Leadership development in nursing, leadership competences, theoretical integration, leadership identity development and curriculum programme.<br>A targeted content analysis was carried out, based on a literature review and theoretical reflection, in order to summarise existing definitions of leadership, conceptualise leadership development capacity and synthesise existing leadership theories.	The study resulted in the proposal of a leadership development model based on 3 main dimensions necessary for leadership development: knowing, doing and being.<br>The dimensions of knowing and doing include 9 leadership competences, inspired by Gardner's tasks (1990).<br>The dimension of being refers to the perception of leadership as an essential part of nursing practice and the identification of the individual as a leader.<br>The context in which the action takes place is understood as the fourth dimension of the model, considered essential and described as contextual intelligence (the ability to assess variables that influence actions and perceptions in the environment). By integrating all these dimensions, it is hoped to promote positive changes, favourable working environments and the provision of quality care.<br>The study concludes that the model offers a conceptual map that structures and facilitates the development of leadership in pre-licence nursing students, guiding the design of curricular content and educational experiences and promoting their integration and application in clinical practice.
**16**	Oh, S & Lim, J.<br>2019<br>South Korea	Developing and evaluating a camp-style leadership enhancement programme for nursing students.	Developing and evaluating a camp-style leadership improvement programme (CLEP) for nursing students, focused on increasing their leadership skills.	Experimental study<br>(pre-test and post-test).	70 second-year nursing students.	The study was carried out at a nursing university and the data was collected between 6 October and 1 December 2015.	Transformational leadership, self-leadership, servant leadership, development of leadership competences, academic training and extracurricular activities.<br>In this study, the Self-Leadership Questionnaire for self-leadership, the Multifactor Leadership Questionnaire for Transformational Leadership and the Servant Leadership Instrument were used as data collection instruments.	The results showed that the experimental group achieved significantly higher scores in self-leadership, transformational leadership and servant leadership compared to the control group. Therefore, it was concluded that CLEP made a significant contribution to the development of leadership skills, proving to be an effective educational approach in stimulating these skills in nursing students.<br>Participants expressed a high level of satisfaction with the programme, which shows that it was well received and considered effective for developing leadership skills.<br>The authors suggest integrating CLEP into students' portfolios as an extracurricular part in order to optimise leadership training. In addition, they also suggest expanding the programme with a view to developing a similar initiative aimed at clinical nurses, focused on the continuous improvement of their leadership skills.
**17**	Öz, G & Abaan, S.<br>2021<br>Turkey	Use of a flipped classroom "leadership in nursing" course on nursing students' achievement and experiences: A quasi-experimental study.	To evaluate the effect of the flipped classroom through the "Leadership in Nursing" course on the results of nursing students (exam grades and learning outcomes).<br>Describe students' experiences of the flipped classroom.	Quasi-experimental study.	39 third-year students who were enrolled in "Leadership in Nursing".	The study was carried out at a university and data collection took place during the autumn semester of the 2017-2018 academic year, over 14 weeks, with 12 weeks dedicated to instruction and 2 weeks to assessment.	Leadership, development of leadership skills, active learning methods, flipped classroom and academic training.<br>Data was collected using various instruments: (1) Student Introductory Information Form; (2) Exams (one mid-term exam, one final exam and 4 tests): (3) Classroom Observation Form; and (4) Student Feedback Form.<br>Quantitative methods were used to assess the students' cognitive progress and their exam grades.<br>In order to collect data on the students' experiences with the Inverted Classroom, interviews were carried out.	The study concluded that the Flipped Classroom approach resulted in high levels of fulfilment of the proposed objectives and higher grades, which confirmed the hypotheses formulated: Students in the Flipped Classroom group were more successful in the course than those in the Traditional Teaching group and also had better learning outcomes.<br>It was observed that students who regularly watched the videos and completed the online learning exercises obtained better results than those who did not.<br>The students in the Flipped Classroom group had access to the course material on the online platform at any time and from anywhere, which encouraged them to take more responsibility for their own learning process.<br>The flipped classroom environment promoted active learning methods, which resulted in greater engagement in class, higher marks and the development of leadership skills.<br>The students highlighted the flexibility and preparation for classes as positive points, as it led them to take responsibility for their learning and made them more proactive and self-confident. The negative aspects identified were the technical/information problems, the requirement for independent and individualised study, the large number of activities and the need for active participation
**18**	Paim, C. et al.<br>2021<br>Brazil	Leadership development of nursing students in a material and sterilisation centre.	To analyse the experiences of nursing students during their compulsory hospital internship in a Material and Sterilisation Centre, with a focus on leadership development.	A descriptive-reflective experience report.	2 nursing students, from the last semester of the nursing course.	The study was carried out as part of a compulsory curricular internship at a general university hospital in southern Brazil between March and June 2019.	Leadership, development of leadership competences and academic and practical training/internship.<br>The students' notes on their internship experiences were analysed.	The authors concluded that the internship in the Material and Sterilisation Centre favoured the development of leadership, management, decision-making and autonomy skills in nursing students. During the internship they had the opportunity to experience the coordination of a unit, which allowed them to develop skills in managing people and materials, conflict resolution, planning and (assertive) communication.<br>The analysis highlighted the importance of integrating and articulating theory and practice, proving that these students' leadership learning took place in concrete and contextualised situations.<br>The trust and sense of belonging to the team reported by the students throughout the internship were highlighted as facilitating factors for the development of leadership in the students' insertion in the Material and Sterilisation Centre, since

								they allowed and encouraged the students to take on leadership behaviours.
**19**	Pardo, M. et al.<br>2021<br>Spain	Fostering leadership competence and satisfaction in nursing undergraduates through a student-eld conference: A quasi-experimental pre-post study.	To evaluate the impact that participation in a student-led conference had on nursing students' self-perception of leadership competence.	Quasi-experimental study, pre-post-test.	31 final year nursing students (enrolled in elective modules) over two academic years (2018-2019 and 2019-2020).	The study was carried out at the Faculty of Medicine and Health Sciences of the Universitat International de Catalunya in the 2018-2019 and 2019-2020 academic years.	Leadership, transformational leadership, development of leadership competences, self-perceived leadership, academic training and scientific conference.<br><br>The Spanish version of the SALI, the ES-SALI, was used to assess the self-perceived leadership of students involved in planning and organising a scientific conference.<br>The level of satisfaction of conference participants was measured using an ad hoc self-completion questionnaire, which consisted of 5 closed-answer questions and 4 open-answer questions relating to suggestions.	Analysing the data collected showed that there was a significant increase in the total score of the ES-SALI and each of the dimensions, although this increase was more marked in the "Impact and Influence" dimension. These results indicate an improvement in the students' perception of leadership competences, highlighting the positive impact of the intervention on skills development.<br>The students said that the experience allowed them to develop skills in organisation, task management, teamwork, communication and research, providing them with an opportunity to grow both professionally and personally.<br>The tutor's contribution throughout the process was considered crucial, providing the supervision and support necessary for the success of the conference, which was a new experience for all of them.<br>Satisfaction with the conference was highly positive for everyone involved.
**20**	Pardo, M. et al.<br>2022<br>Spain	Enhancing perceived leadership of nursing students through a student-led dedicated education unit in a community setting: A feasibility study.	To demonstrate the feasibility of increasing perceived leadership competence among nursing students through participation in an innovative strategy of health education.	Feasibility study examining the acceptability, implementation and subsequent expansion of a student-led Dedicated Education Unit (DEU) in the field of health education.	31 nursing students from the third and fourth-year of the BSN programme.	The study was developed and tested in a health centre in Barcelona between September 2014 and July 2019, through a three-phase process. Data was collected at phase II, which took place in the academic years 2017-2018 and 2018-2019, during a clinical internship.	Leadership, clinical leadership, development of leadership competences, perception of leadership competences, student-led dedicated teaching units (DEU) and practical training/internships.<br><br>In order to assess the acceptability and implementation of the DEU, three instruments were drawn up: a sociodemographic questionnaire aimed at students and two satisfaction questionnaires, one aimed at students and the other at service users.<br>The students' self-perception of leadership competence was assessed using the Spanish version of the Self-Assessment Leadership Instrument (ES-SALI).	The analysis of the data showed a significant increase in nursing students' perception of leadership competences after the internship, both in the intervention group (assigned to the DEU) and in the control group (students doing a normal community health internship in a health centre). However, there was a more significant increase in the intervention group, with significant improvements in the Impact and Influence and Teamwork Skills dimensions.<br>The DEU was highly acceptable, with high satisfaction ratings from students and service users.<br>The students' self-assessments were higher than the mentors' assessments of the students' leadership competences, confirming previous findings.<br>The DEU has been shown to be applicable in different socio-cultural contexts, suggesting its potential for international transfer, although more research in this area is recommended.
**21**	Putra, A. et al.<br>2021<br>Indonesia	How is the leadership behaviour of undergraduate nursing students in Aceh? A cross sectional study.	To determine the level of development of leadership behaviour in students of the Faculty of Nursing, Universitas Syiah Kuala (FON-USK), who applied the CBC with various learning methods in the implementation process.	Cross-sectional descriptive study.	382 nursing students.	The study was carried out at the Faculty of Nursing at Universitas Syiah Kuala in Banda Aceh, Indonesia. The data was collected in May 2018.	Leadership, development of leadership competences/behaviours, academic training, curriculum, competence-based curriculum (CBC), active learning methods, role-play and simulation (RPS), collaborative learning (CbL), problem-based/inquiry-based learning (PBL/I) and self-directed learning (SDL).<br><br>An instrument in the form of a questionnaire was used, with one part relating to demographic data and the other to the Pengembangan Perilaku Kepemimpinan Mahasiswa/Student Leadership Behaviour Development (PPKM/SLBD) questionnaire.	The study highlights the importance of developing leadership skills in the teaching-learning process, emphasising the need to train competent nurses who can take on leadership roles in the nursing profession.<br>The results obtained indicate that the leadership behaviour of FON-USK students was in the "good" category, with 94.2% of participants being rated positively. These data suggest that the implementation of the Competency-Based Curriculum (CBC), combined with the various learning methods used (roleplay and simulation; collaborative learning; problem-based/inquiry-based learning; and self-directed learning), was effective in promoting leadership competences among nursing students.<br>It is concluded that the use of diversified teaching methods such as Student-Centred Learning (operationalised in this study by the CBC) contributes significantly to the development of these competences, preparing students to face future challenges.
**22**	Reime, M. et al.<br>2022<br>Norway	Baccalaureate nursing students' reflections on professional development within the nursing leadership role when participating in student-run teams: A qualitative content analysis of student's reflection notes.	To explore nursing students' reflections on the development of nursing leadership competence when they participate in student-run teams (SRT).	A qualitative, descriptive and exploratory study.	37 second-year nursing students.	The study was carried out at a large university hospital in western Norway during a student internship. No other information regarding the data collection period was made available.	Leadership, development of leadership skills, student-run teams (SRT), critical reflection, safe and authentic clinical environment, professional identity and academic and practical/internship training.<br><br>An inductive and qualitative content analysis was carried out on the 37 reflective notes collected.	Analysing the students' reflections revealed a main theme: Leadership competences in nursing are developed in an authentic and safe clinical environment. The sub-themes identified were:<br>• The discovery and recognition of the leadership role contributed to the development of the nursing identity: The students recognised that the development of the nursing identity was acquired through guidance in authentic and safe clinical situations in practice;<br>• Being challenged and taking on responsibilities in the SRT contributed to the development of leadership skills in nursing;<br>• The way services are organised affects the quality of care and satisfaction with one's work situation.<br>The students emphasised the need for more training in nursing leadership and recognised the importance of continuity, both of care and of leadership, to improve the quality of care and satisfaction with the practice environment.<br>The SRT experience prepared students to take responsibility for groups of patients and colleagues in the transition to professional practice. Their active participation contributed to the strengthening of nursing identity, allowing the essential aspects of the leadership role to be experienced and understood
**23**	Sarnkhaowkhom, C. et al.<br>2022<br>Thailand	Assessment of entrepreneurial leadership among undergraduate nursing students: The case from Thailand.	To explore entrepreneurial leadership and examine its associated factors among undergraduate nursing students.	Cross-sectional study.	410 nursing students, from the first to the fourth-year of the nursing degree course, in the 2020 academic year.	The study was carried out at a private university in Bangkok and the data was collected between October and November 2020.	Leadership, innovation, entrepreneurship, entrepreneurial leadership and leadership competences.<br><br>The data was collected using an entrepreneurial leadership questionnaire developed by the researchers, made up of 36 items, divided into 4 components: personal competence, management competence, proactive competence and technological competence.<br>A questionnaire was also administered regarding demographic data: gender, age, year in school, grade point average, parents' income,	A high level of entrepreneurial leadership was obtained in the students under study.<br>Technological competence was the component with the highest score.<br>Age, curriculum year and parental income were considered critical factors influencing the development of entrepreneurial leadership.<br>Entrepreneurial approaches have the potential to improve all aspects of students' education and should therefore be utilised.<br>Students should learn and practice entrepreneurial leadership behaviours to develop their professional skills and facilitate the innovation process.<br>Future research is suggested to identify strategies that promote the development of entrepreneurial leadership skills in nursing students, as well as technological competence and the implementation of new health technologies.

							parents' expenses, part-time work and type of part-time work.	
**24**	Spigelmyer, P. & Loughran, M.<br>2022<br>United States of America	Simulation: An active learning pedagogy for an undergraduate nursing leadership course.	To evaluate the use of low-fidelity simulation together with interpretive pedagogy in a leadership and management course in nursing (from a BSN curriculum) as an effective active learning strategy to improve critical thinking skills as well as quality and safety knowledge and competences among nursing students.	Educational intervention study.	117 undergraduate nursing students taking the leadership and management course.	The study was carried out at an urban private Catholic university. Data collection took place in April 2018.	Leadership, development of leadership skills, active learning method, simulation, interpretive pedagogy, TeamSTEPPS and academic training.<br><br>A TeamSTEPPS Learning Benchmarks test (a 15-item instrument used to assess student learning and critical thinking) was used. It was applied before and after the simulation.<br>A questionnaire with qualitative questions was also used to gather information on the students' perceptions and opinions of the simulation event.	The study concludes that simulation, as an alternative clinical experience, is an effective strategy for providing students with exposure to realistic clinical contexts that favour cognitive and affective learning, as well as the development and strengthening of leadership skills. In this study, the use of this methodology supported the students' learning process and was enhanced by the preparatory activities that were carried out before the simulation.<br>The results obtained indicate that the integration of an interpretive pedagogical approach improved the students' ability to critically reflect on their interventions, allowing them to adjust their decisions in real contexts and in different clinical practice scenarios.<br>Analysing the data highlighted the need to further train students in essential areas such as patient safety and assertiveness. Clinical trainers play a vital role in this process, and it is imperative that they pay attention to the clinical environment and encourage students' actions in defence of their patients, especially on safety issues.<br>Simulation has proved to be a cost-effective way of giving students clinical experience, especially when it is not possible to do so in the healthcare system.<br>The students expressed positively that they enjoyed the simulation experience and recognised the benefits of the day.
**25**	Stubin, C. et al.<br>2024<br>United States of America	Addressing the 2021 *Essentials* with new approaches for developing leadership, resilience, and self-care/well-being in undergraduate baccalaureate nursing students.	Demonstrate how the School of Nursing has implemented approaches to prepare the next generation of nurses with strong competences in the areas of leadership, resilience and self-care/wellbeing, while addressing *The Essentials*.	Pilot study/pilot project.	Approximate cohort of 45 level four students (final semester).	The study was carried out at a university nursing school and the data collected during the spring semester of 2023.	Development of leadership, resilience and self-care/well-being skills, AACN *Essentials*, curricular programme and academic and practical/internship training.<br><br>The measurement instruments used were not specified. There is only reference to informal evaluations by teachers of the activities carried out.	Most of the teaching strategies were easily integrated into the existing curriculum, having been incorporated during the designated class time, and the results showed that they contributed to the development of leadership, resilience and self-care/well-being skills in the students.<br>The students' comments indicated high levels of satisfaction with the strategies used. However, some felt overwhelmed by the amount of information and activities related to the strategies, which suggests the need to balance the workload.<br>The authors recommend that the strategies be scheduled during class time and clinical teaching to maximise student participation. They also suggest introducing these concepts from the first levels of the nursing curriculum and continuing them throughout all levels.<br>The study concludes that the initiative is sustainable, as most of the strategies implemented involved little or no cost and were supported by collaborations with other organisations.<br>The focus on leadership skills, resilience and self-care/well-being is essential for preparing future nurses, helping to prevent burnout and promote effective nursing practices.
