# Supplementary material for: Leadership Development in Undergraduate Nursing Students: A Scoping Review
Source: Nurs Rep. 2025 May 2;15(5):160. doi: 10.3390/nursrep15050160 (PMC12114338; doi:10.3390/nursrep15050160)
Supplement: Supplementary file 1 [file nursrep-15-00160-s001.zip › Supplementary File S3_Synthesis by meta-aggregation.pdf]

<u>Supplementary File S3</u>: Synthesis by meta-aggregation

Finding	Category	Synthesised finding
• <u>Recognising leadership as an essential competence</u>:<br>○ Leadership was widely recognised by students as an essential skill for nursing practice [38, 56, 61, 72, 73].<br>○ Leadership directly influences NPEs, the quality and safety of the care provided, the resolution of clinical complications, satisfaction, motivation and teamwork, as well as creating models for others to follow [38, 73].<br>○ Ineffective leadership can lead to errors and adverse effects, affecting patient safety and health outcomes, which can be minimised by effective leadership [38].<br>• <u>Lack of preparation of students to lead:</u><br>○ Despite recognising the importance of leadership, the majority of students said they did not feel prepared to take on this role [38, 72].<br>○ 70% of students admitted that they did not feel prepared to lead nursing teams in the future and recognised the urgent need to develop skills in this area [38].<br>○ The majority of students report engaging in leadership behaviours, but they warn of the need and importance of ensuring more consistent and structured training and application throughout undergraduate training, in order to promote solid and consistent development [72].<br>• <u>Clinical leadership as an essential tool:</u><br>○ Clinical leadership has been identified as crucial for integrating theoretical knowledge into practice and for promoting safer and more effective care [58,61,73].<br>○ The students considered it a fundamental need for personal development and for improving the organisation and definition of priorities in care [73].<br>• <u>Integrating leadership into curricula:</u><br>○ There is a consensus that the teaching of leadership should be integrated into the curricula of undergraduate training from the earliest years and developed in a continuous and consistent way, in conjunction with various disciplines [1,27,29,38,53-73].<br>○ Updating curricula is seen as essential, as many are criticised for being excessively focused on technical skills, which limits the development of a holistic view of nursing [38, 56].<br>○ The lack of specific lessons on leadership was identified by 40 per cent of the students in Aydogdu's study [38], who had not received this type of training, even after completing more than half of the course.<br>• <u>Recommendations for a more effective approach to teaching leadership:</u><br>○ A greater focus on dynamic methodologies has been suggested, such as realistic simulations, educational videos, theoretical-practical classes, events on leadership and reflective practices [38,56,57].<br>○ The importance of the continuous link between theory and practice has been emphasised, with practical training playing an essential role in the development of leadership [58,63,73].<br>• <u>The role of the teacher in developing leaders:</u><br>○ Teachers are seen as responsible for preparing students for leadership, and should prioritise practical experiences and be role models for students [57,62].<br>○ The qualified intervention of teachers is considered crucial to the learning process, where they must identify opportunities to exercise leadership in a controlled and safe environment [57, 61].	**Students' perception of leadership**	The students recognised leadership as an indispensable skill for nursing practice, with a direct impact on the quality and safety of the care provided. However, they also expressed a general perception of insufficient preparation to take on this role, highlighting an urgent need to restructure curricula so that they integrate its teaching from the initial years of training.
• Tutoring/Mentoring [53];<br>• Realistic simulation [54,66];<br>• Flipped classroom [64];<br>• Student-led conferences [27];<br>• Student-run teams [71];<br>• Camp-style Leadership Education Programme [67];<br>• Dedicated Teaching Unit (DEU) [59].	**Strategies to train leaders in nursing**	The implementation of innovative methods and approaches to promote leadership development in nursing students seems essential to ensure the training of effective leaders.
• The Self-Assessment Leadership Instrument (SALI) scale for the Spanish cultural context (ES-SALI) [29];<br>• The Educational Leadership Scale for Nursing Students [65];<br>• The Nursing Leadership Competence Assessment Scale for Undergraduate Nursing Students (NLCAS/UNS) [70];<br>• The Pengembangan Perilaku Kepemimpinan Mahasiswa/Student Leadership Behaviour Development (PPKM/SLBD) [68];<br>• The Self-Leadership Questionnaire [67];<br>• The Schwarzer and Jerusalem's General Self-Efficacy Scale (GSES) [86], combined with a questionnaire developed by the researchers [66];<br>• The Entrepreneurial Leadership of Nursing Students Questionnaire [69];<br>• 15-item tool from the TeamSTTEPS Learning Benchmarks to assess the impact of simulation on the development of leadership in students [54].	**The evaluating leadership development**	Integrating these tools into undergraduate training has a significant impact on the development of competences, promoting dynamic and reflective learning. They also make it easier to personalise teaching interventions, allowing areas for improvement to be identified and strategies to be adjusted for continuous progress.
• The Leadership Development Model by Miles and Scott [1];<br>• Innovative Objectives for Leadership Development [70];<br>• Implementation of a Competence-Based Curriculum (CBC) [68];<br>• The *Essentials* of the American Association of Colleges of Nursing [55];<br>• Continuous integration between theory and practice, with clinical leadership promoting this process [62];<br>• Conceptual model for the development of clinical leadership in undergraduate nursing programmes [55];<br>• Integration of content in a structured way throughout the curriculum, relating to three main dimensions: knowledge, skills and behaviour [61].	**Conceptual models and curricular programmes**	The integration of the teaching and development of leadership must be done in a structured, continuous and transversal way, recognising its importance not only as a competence, but also as a value.
